# Bioinformatics analysis reveals immune prognostic markers for overall survival of colorectal cancer patients: a novel machine learning survival predictive system

**DOI:** 10.1186/s12859-022-04657-3

**Published:** 2022-04-08

**Authors:** Zhiqiao Zhang, Liwen Huang, Jing Li, Peng Wang

**Affiliations:** Department of Infectious Diseases, Shunde Hospital, Southern Medical University, Shunde, Guangdong China

**Keywords:** Colorectal cancer, Immune gene, Prognostic model, Overall survival

## Abstract

**Objectives:**

Immune microenvironment was closely related to the occurrence and progression of colorectal cancer (CRC). The objective of the current research was to develop and verify a Machine learning survival predictive system for CRC based on immune gene expression data and machine learning algorithms.

**Methods:**

The current study performed differentially expressed analyses between normal tissues and tumor tissues. Univariate Cox regression was used to screen prognostic markers for CRC. Prognostic immune genes and transcription factors were used to construct an immune-related regulatory network. Three machine learning algorithms were used to create an Machine learning survival predictive system for CRC. Concordance indexes, calibration curves, and Brier scores were used to evaluate the performance of prognostic model.

**Results:**

Twenty immune genes (BCL2L12, FKBP10, XKRX, WFS1, TESC, CCR7, SPACA3, LY6G6C, L1CAM, OSM, EXTL1, LY6D, FCRL5, MYEOV, FOXD1, REG3G, HAPLN1, MAOB, TNFSF11, and AMIGO3) were recognized as independent risk factors for CRC. A prognostic nomogram was developed based on the previous immune genes. Concordance indexes were 0.852, 0.778, and 0.818 for 1-, 3- and 5-year survival. This prognostic model could discriminate high risk patients with poor prognosis from low risk patients with favorable prognosis.

**Conclusions:**

The current study identified twenty prognostic immune genes for CRC patients and constructed an immune-related regulatory network. Based on three machine learning algorithms, the current research provided three individual mortality predictive curves. The Machine learning survival predictive system was available at: https://zhangzhiqiao8.shinyapps.io/Artificial_Intelligence_Survival_Prediction_for_CRC_B1005_1/, which was valuable for individualized treatment decision before surgery.

**Supplementary Information:**

The online version contains supplementary material available at 10.1186/s12859-022-04657-3.

## Introduction

The latest research showed that colorectal cancer (CRC) was the fourth most common cancer in the world, resulting in 1,096,601 new cases and 551,269 deaths in 2018 [1]. Although great progress has been made in diagnosis and treatment of CRC, global data demonstrated that the mortality was still unsatisfactory for CRC patients [2]. Alterations of chromosomal copy number, gene methylation, and gene expression were involved in the occurrence and progress of CRC, leading to huge heterogeneity of prognosis in CRC patients [3, 4]. Due to the huge demand for predicting the prognosis of patients with colorectal cancer, different research teams have established prognostic models for patients with colorectal cancer based on different prognostic markers [5–7]. However, the calculation formulas of these exquisite prognostic models are complex, which seriously restricts the popularization and application of clinical practice. Due to the huge heterogeneity of prognosis in CRC patients, a single biomarker was not enough to provide accurate prognostic information for CRC patients. More importantly, most of the current prognostic models could only predict the prognosis for a special group, but could not predict the prognosis for an individual patient [8, 9]. From the patient's point of view, mortality risk predicted percentage for an individual patient is more valuable and important than that for a special group. Therefore, it is necessary and valuable to construct predictive models for providing individual mortality risk prediction.

A large number of molecular biological evidences have confirmed that genes played important roles in the endogenous regulation of tumorigenesis and progression [10–13]. Immune microenvironment was closely related to tumor development, progression and prognosis [14, 15]. Several studies have explored the potential roles of immune genes in the prognosis of CRC [16–18]. Two immune-related prognostic models were developed for predicting prognosis of CRC patients [19, 20]. Hu et al. established a prognostic model of colorectal cancer through CEACAM8+ neutrophils, CD3+, CD8+ T lymphocytes and FOXP3 + regulatory T cells [19]. Zhou et al. established a prognostic immune risk score for stage I–III colon cancer patients with an area Under the receiver operating characteristic curve of 0.741 in train dataset for 5-year mortality [20]. However, these two models failed to provide individual mortality risk prediction for a specific patient.

Machine learning has been applied to medical image recognition, diagnosis and prognosis [21, 22]. Kawakami et al. used different machine learning algorithms to predict the clinical stage and pathological type for ovarian cancer patients [23]. Enshaei et al. created an machine learning model to predict the prognosis of ovarian cancer patients [24]. These studies provided new insights for the applications of machine learning in diagnosis and prediction. However, to date, there is no clinical study on machine learning model for predicting the individualized mortality risk for various tumors.

Our research team was committed to develop precision medical predictive tools for predicting the individualized mortality risk for different tumors [25–32]. Inspired by the above machine learning researches, we planned to build and verify an machine learning survival predictive system to predict the individual mortality risk based on machine learning algorithms and immune genes for CRC patients.

## Methods

### Study datasets

TCGA dataset involved 20,236 mRNAs and 521 CRC patients. The original expression values were log2 transformed. GSE39582 dataset involved 556 CRC patients and 23,494 mRNAs [33]. Probe IDs were generated on GPL570 platform and gene symbols were determined by Gencode.v29. Flow chart (Additional file 5: Fig. S1) displayed the flow chart of the current study. For survival analysis, GSE39582 dataset was used as model dataset and TCGA dataset was used as validation dataset.

### Differentially expressed analyses

Differentially expressed analyses were performed between 480 tumor samples and 41 normal samples. Log_2_ |fold change|> 1 and *P* value < 0.05 were defined as cut off values. Package “edgeR” was used to normalize the original expression values with Trimmed mean of M values method [34].

### Immune gene

Immune genes were determined in Immunology Database and Analysis Portal database [35]. Cistrome Cancer database was used to search transcription factors [36]. To screen transcription factors highly related with immune genes, |correlation coefficient|> 0.5 and *P* value < 0.01 were defined as cut-off values. Gene biological processes were identified through TISIDB database. Tumor immune infiltration indexes were calculated through single sample gene set enrichment analysis [37, 38].

### Introduction of regression algorithms

The prediction of mortality risk based on individual level is helpful to optimize the level of individualized treatment for cancer patients. In order to provide the mortality probability of a special individual patient at all time points, some extended regression algorithms, including Cox proportional hazard regression model, Random Survival Forest model, and Multi-Task Logistic Regression model, were used to provide individual mortality risk curves of cancer patients [39].

### Cox proportional hazard regression algorithm

Cox proportional hazard regression model was carried out according to the original articles [40, 41]. The advantage of Cox proportional hazards regression analysis is that it can be applied to both measurement variables and classification variables. Meanwhile, Cox proportional hazards model can simultaneously show the impact of multiple independent variables on survival outcome.

### Random survival forest algorithm

Random survival forest is an integrated algorithm based on the combination of multiple decision trees with the following advantages: handling capacity of non-linear effect; evaluation of variable relative importance and selection of important variables according to the given threshold; exploration of the relationship between included variables and study outcomes [42, 43]. Based on the samples in original cohort, bootstrap method was used to construct a lot of new trees for training the random survival forest [44]. For each branch node, the best combination of variables used to split the branch is generated based on the principle of maximizing the difference between the next branch groups. Random survival forest has been used in clinical research and showed good application ability in variable selection and outcome prediction [43–46].

### Multi-task logistic regression algorithm

Multi-task logistic regression (MTLR) has been proposed for clinical medicine through combining multiple logistic regression models in a dependent way to establish a predictive function [47]. MTLR model can be used to predict the survival probability of an individual in a certain time range. MTLR model was superior to logistic regression model in goodness of fit and prediction performance [48]. Other details of machine learning algorithms could be found in our previous studies [25, 27–32, 49].

### Statistical analyses

Statistical analyses were carried out by SPSS Statistics 19.0 (SPSS Inc., USA). Machine learning and bioinformatics analyses were performed by Python language and R software language with appropriate packages and corresponding algorithms [25, 27–32, 49]. The top important packages included pec, rms, survival, rmda, ggplot2, GOplot, timereg, randomForestSRC, and riskRegression.

## Results

### Study datasets

Table 1 displayed clinical features of CRC patients. Ninety-eight patients out of 428 patients died in TCGA dataset (validation) and 187 patients out of 556 patients died in GSE39582 dataset (model dataset).

**Table 1:** Clinical features of included patients

	TCGA	GSE39582	*P* value
Number (n)	428	556	
Death [n(%)]	98(22.9)	187(33.6)	< 0.001
Total survival time (mean ± SD, month)	29.8 ± 25.6	57.5 ± 38.3	< 0.001
Survival time for dead patients (month)	23.3 ± 22.7	37.4 ± 28.9	< 0.001
Survival time for living patients (month)	31.7 ± 26.1	67.6 ± 38.4	< 0.001
Age (mean ± SD, year)	66.5 ± 13.0	66.7 ± 13.3	0.825
Male [(n)%]	230(53.7)	306(55.0)	0.686
Stage 4 [n(%)]	60(14.0)	59(10.6)	< 0.001
Stage 3 [n(%)]	124(28.9)	203(36.5)	
Stage 2 [n(%)]	163(38.1)	258(46.4)	
Stage 1 [n(%)]	70(16.4)	31(5.6)	
Stage (NA) [n(%)]	11(2.6)	4(0.7)	
AJCC PT (T4) [n(%)]	51(11.9)	117(21.0)	< 0.001
AJCC PT (T3) [n(%)]	294(68.7)	360(64.7)	
AJCC PT (T2) [n(%)]	72(16.8)	43(7.7)	
AJCC PT (T1) [n(%)]	11(2.6)	12(2.2)	
AJCC PT (NA) [n(%)]	0	24(4.3)	
AJCC PN (N2) [n(%)]	76(17.8)	104(18.7)	< 0.001
AJCC PN (N1) [n(%)]	103(24.1)	131(23.6)	
AJCC PN (N0) [n(%)]	249(58.2)	294(52.9)	
AJCC PN (NA) [n(%)]	0	27(4.9)	
AJCC PM (M1) [n(%)]	107(25.0)	60(10.8)	< 0.001
AJCC PM (M0) [n(%)]	315(73.6)	473(85.1)	
AJCC PM (NA) [n(%)]	6(1.4)	23(4.1)	
Lymphovascular invasion (yes/no/NA)	148/237/43	NA	
Vascular invasion (yes/no/NA)	89/281/58	NA	
Residual tumor (3/2/1/0/NA)	23/21/4/307/73	NA	
Perineural invasion (yes/no/NA)	45/126/257	NA	

Continuous variables were presented as mean ± standard deviation

Continuous variables were compared by t-test or the Kruskal–Wallis H test. Categorical variables were compared by the chi-squared test or Fisher’s exact test

NA, missing data; AJCC, American Joint Committee on Cancer

### Differentially expressed analyses

There were 4087 mRNAs identified by differentially expressed analyses in TCGA cohort. Meanwhile, there were 3588 immune genes identified in TCGA cohort. A total of 1384 differentially expressed immune genes were found after intersecting the datasets of differentially expressed genes and immune genes. Volcano chart (Additional file 5: Fig. S2A) identified 1384 differentially expressed immune genes (779 up-regulation and 605 down-regulation).

### Functional enrichment analyses

**Fig. 1:**
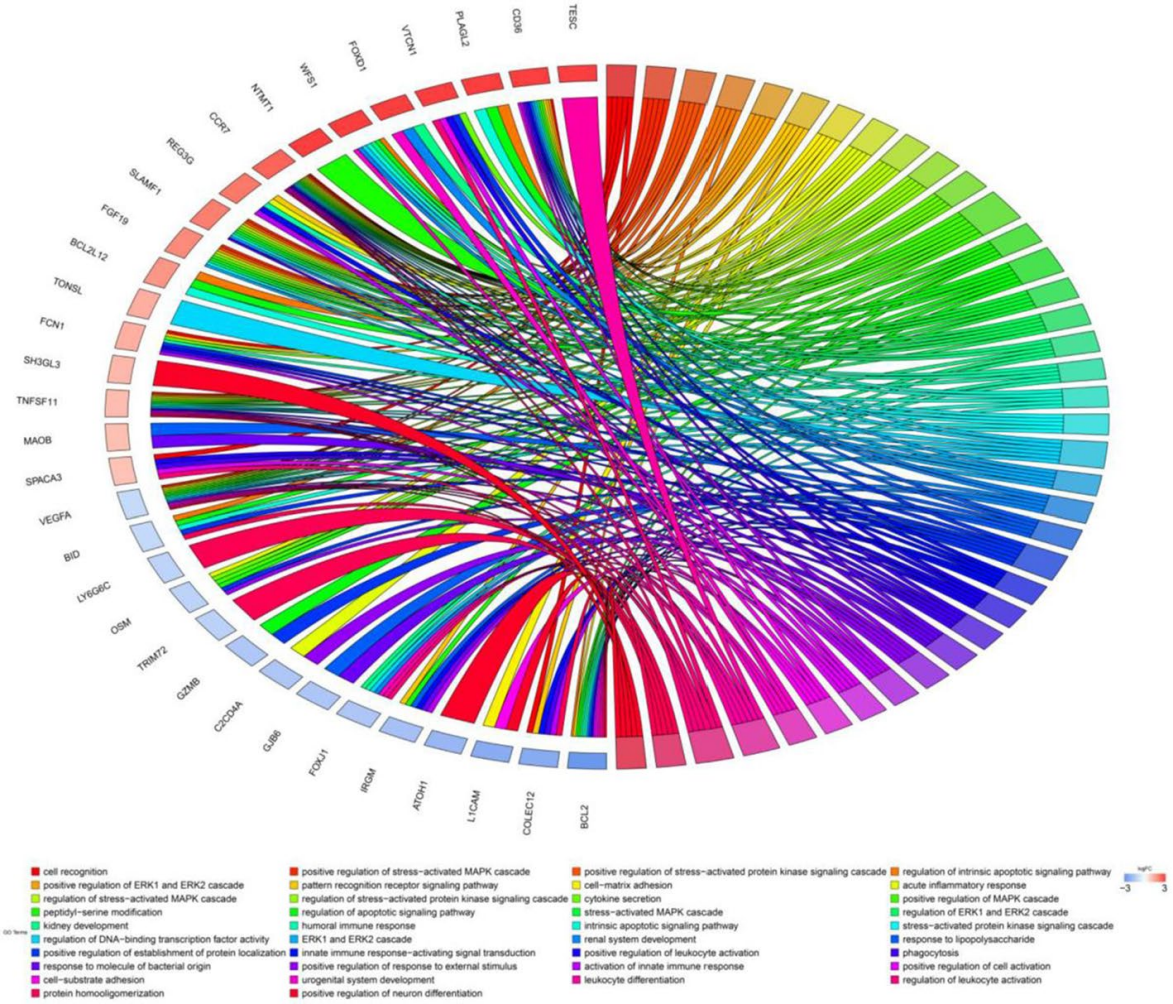
Chord chart of immune genes

Gene Ontology chord chart (Fig. 1) and Bar chart (Additional file 5: Fig. S2B) showed that biological processes of immune genes were mainly enriched in: positive regulation of MAPK cascade, regulation of apoptotic signaling pathway, regulation of DNA-binding transcription factor activity, positive regulation of establishment of protein localization, leukocyte differentiation, regulation of leukocyte activation, cell recognition, positive regulation of stress-activated MAPK cascade, positive regulation of stress-activated protein kinase signaling cascade, and regulation of intrinsic apoptotic signaling pathway.

### Immune regulatory network

**Fig. 2:**
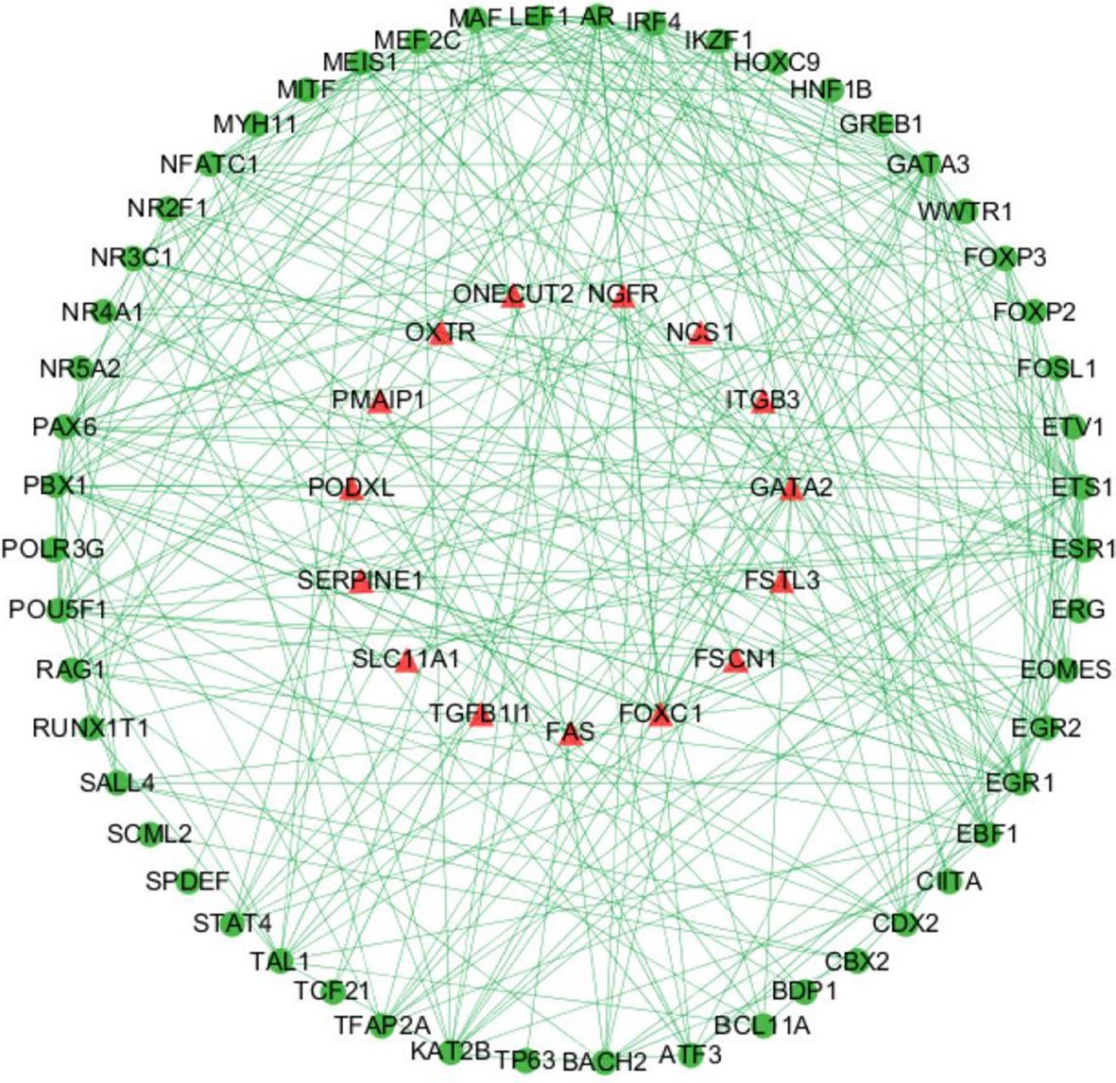
Immune genes regulatory network chart. *Note*: The red triangle represents the transcription factor and the green circle represents the immune gene

The original gene expression values were translated into '1' (as high expression) and '0' (as low expression) according to median values for both GSE39582 dataset and TCGA dataset. Univariate Cox regression identified 119 immune genes as prognostic biomarkers for overall survival (OS). Transcription factors that highly related with prognostic immune genes were identified according to previous thresholds. The associations among immune mRNAs and transcription factors were determined in STRING database. The regulatory network among immune genes and transcription factors was depicted by cytoscape v3.6.1 (Fig. 2).

### Variable selection process

**Fig. 3:**
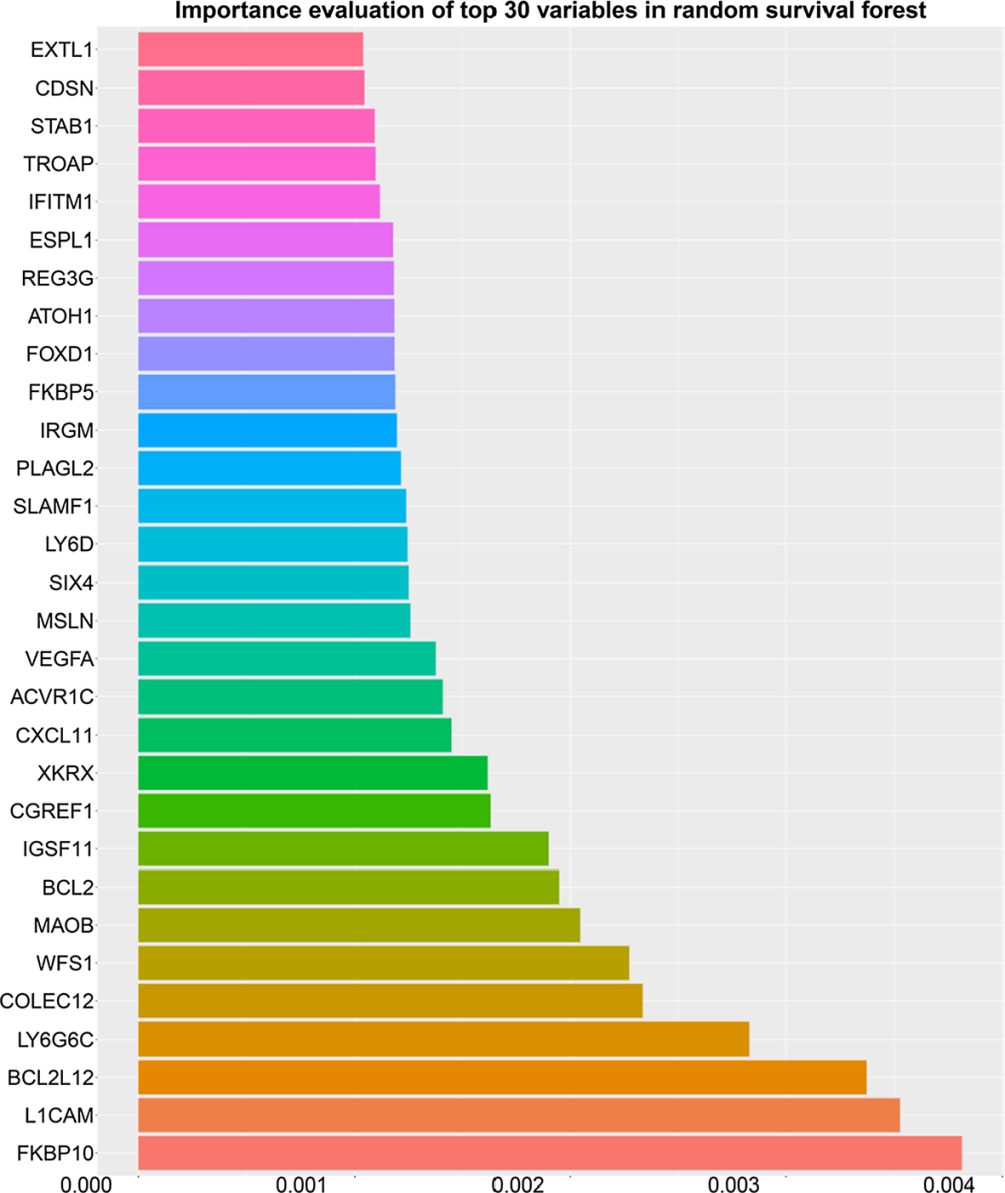
Variable importance assessment chart in random survival forest

The current study first explored the relative importance of different independent variables through the random survival forest package. The top 30 important prognostic immune genes were displayed in Fig. 3. We puted the genes with potential prognostic value found in the random survival forest into the multivariate Cox proportional hazard regression model to further investigate the independent prognostic risk factors of tumor patients. Through the step-by-step iterative method of multivariate COX proportional hazard regression, we explored and ascertained the optimal prognostic model with the highest C index among different gene combinations. The final machine learning survival predictive system was established based on these prognostic genes in optimal prognostic model by using different machine learning algorithms.

### Construction of prognostic model

**Fig. 4:**
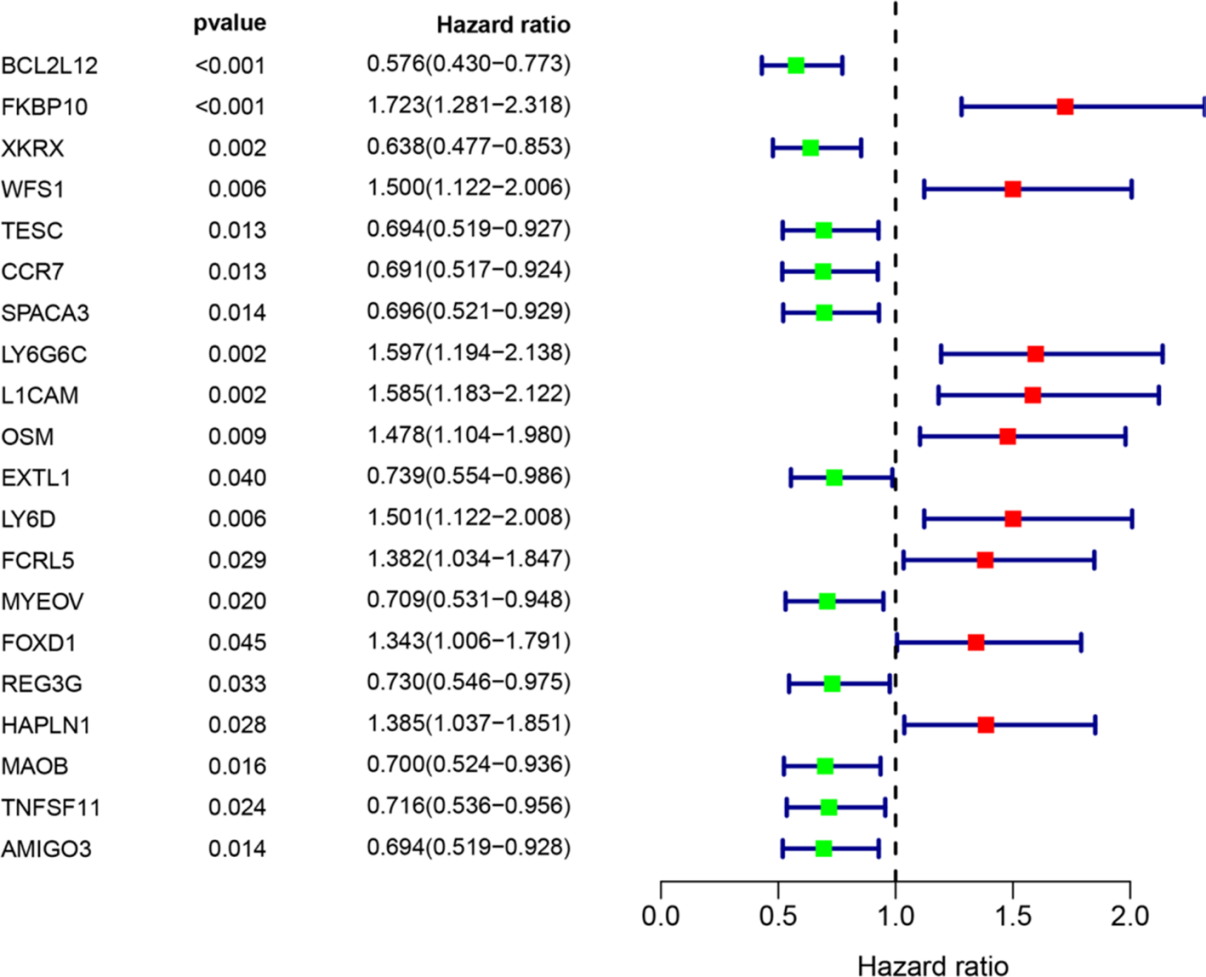
Immune gene survival forest chart

Multivariate Cox regression identified twenty independent prognostic mRNAs for OS (Table2; Fig. 4). The formula of prognostic model was as following: Prognostic score = (− 0.542 * BCL2L12) + (0.479 * FKBP10) +

**Table 2:** Information of prognostic immune genes

Immune gene	Univariate analysis		*P* value	Multivariate analysis			*P* value
	HR	95% CI		Coefficient	HR	95% CI	
BCL2L12 (high/low)	0.576	0.430–0.773	0.001	− 0.542	0.581	0.424–0.798	0.001
FKBP10 (high/low)	1.723	1.281–2.318	0.001	0.479	1.615	1.169–2.231	0.004
XKRX (high/low)	0.638	0.477–0.853	0.002	− 0.347	0.707	0.520–0.961	0.027
WFS1 (high/low)	1.500	1.122–2.006	0.006	0.597	1.817	1.320–2.503	0.000
TESC (high/low)	0.694	0.519–0.927	0.013	− 0.768	0.464	0.328–0.657	0.000
CCR7 (high/low)	0.691	0.517–0.924	0.013	− 0.739	0.478	0.348–0.656	0.000
SPACA3 (high/low)	0.696	0.521–0.929	0.014	− 0.624	0.536	0.390–0.736	0.000
LY6G6C (high/low)	1.597	1.194–2.138	0.002	0.628	1.873	1.374–2.553	0.000
L1CAM (high/low)	1.585	1.183–2.122	0.002	0.530	1.699	1.248–2.314	0.001
OSM (high/low)	1.478	1.104–1.980	0.009	0.709	2.031	1.457–2.832	0.000
EXTL1 (high/low)	0.739	0.554–0.986	0.040	− 0.460	0.631	0.458–0.870	0.005
LY6D (high/low)	1.501	1.122–2.008	0.006	0.602	1.826	1.329–2.508	0.000
FCRL5 (high/low)	1.382	1.034–1.847	0.029	0.583	1.791	1.316–2.438	0.000
MYEOV (high/low)	0.709	0.531–0.948	0.020	− 0.527	0.590	0.433–0.805	0.001
FOXD1 (high/low)	1.343	1.006–1.791	0.045	0.618	1.856	1.350–2.552	0.000
REG3G (high/low)	0.730	0.546–0.975	0.033	− 0.389	0.678	0.491–0.936	0.018
HAPLN1 (high/low)	1.385	1.037–1.851	0.028	0.433	1.542	1.133–2.099	0.006
MAOB (high/low)	0.700	0.524–0.936	0.016	− 0.472	0.624	0.458–0.850	0.003
TNFSF11 (high/low)	0.716	0.536–0.956	0.024	− 0.439	0.645	0.475–0.875	0.005
AMIGO3 (high/low)	0.694	0.519–0.928	0.014	− 0.425	0.654	0.476–0.899	0.009

**Fig. 5:**
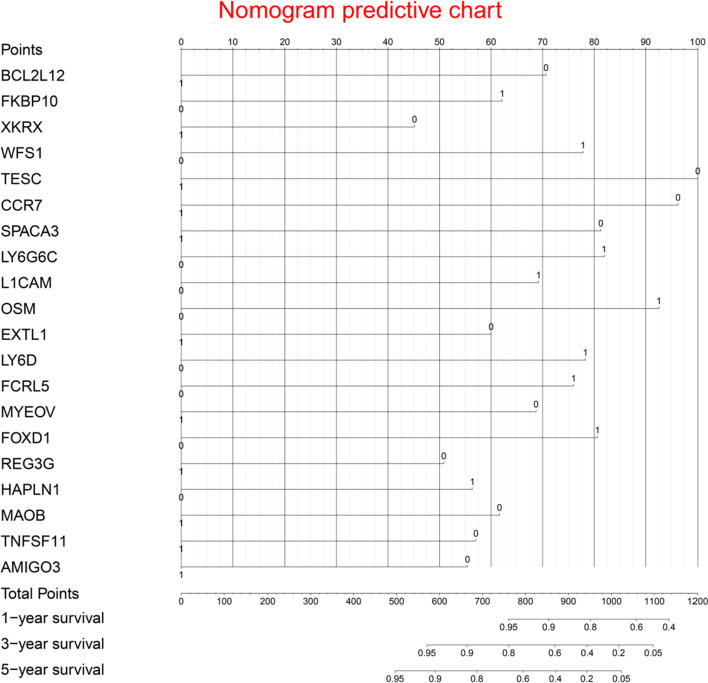
Prognostic nomogram chart

HR, hazard ratio; CI, confidence interval

(− 0.347 * XKRX) + (0.597 * WFS1) + (− 0.768 * TESC) + (− 0.739 * CCR7) + (− 0.624 * SPACA3) + (0.628 * LY6G6C) + (0.530 * L1CAM) + (0.709 * OSM) + (− 0.460 * EXTL1) + (0.602 * LY6D) + (0.583 * FCRL5) + (− 0.527 * MYEOV) + (0.618 * FOXD1) + (− 0.389 * REG3G) + (0.433 * HAPLN1) + (− 0.472 * MAOB) + (−  0.439 * TNFSF11) + (− 0.425 * AMIGO3). A prognostic nomogram was showed in Fig. 5. Therefore RFS model, MTLR model, and Cox model were all based on the previous 20 independent prognostic genes.

Additional file 5: Fig. S3 showed there were significant differences between survival curves of two subgroups for twenty immune mRNAs. Additional file 5: Fig. S4 and Fig. S5 were predictive value distribution chart and survival status scatter chart performed by ggplot2 package, indicating that CRC patients with high prognostic scores tend to have a shorter survival time.

### Performance of cox model in model cohort

**Fig. 6:**
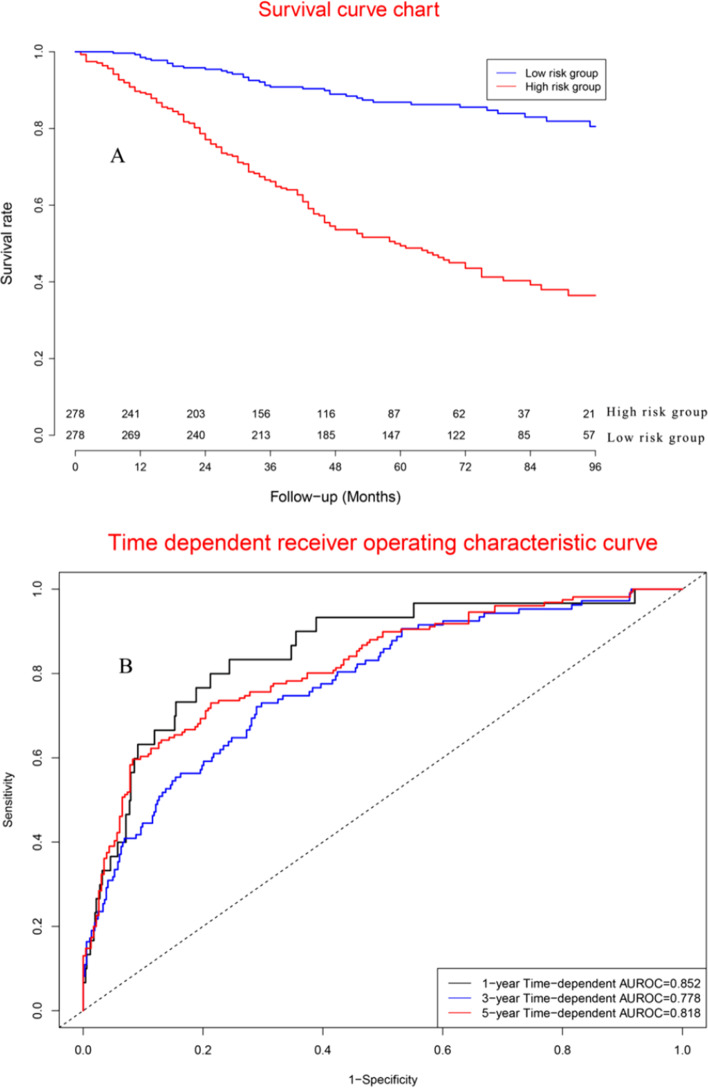
Clinical performance in model cohort: **a** Survival curves for high risk group and low risk group; **b** Time-dependent receiver operation characteristic curves

Survival curve chart (Fig. 6A) indicated that there were significant differences between two groups for prognostic model. Concordance indexes were 0.852, 0.778, and 0.818 for 1-year, 3-year, and 5-year survival (Fig. 6B). Calibration curves (Additional file 5: Fig. S6) showed good agreements between predicted mortality and actual mortality.

### Performance of cox model in validation cohort

**Fig. 7:**
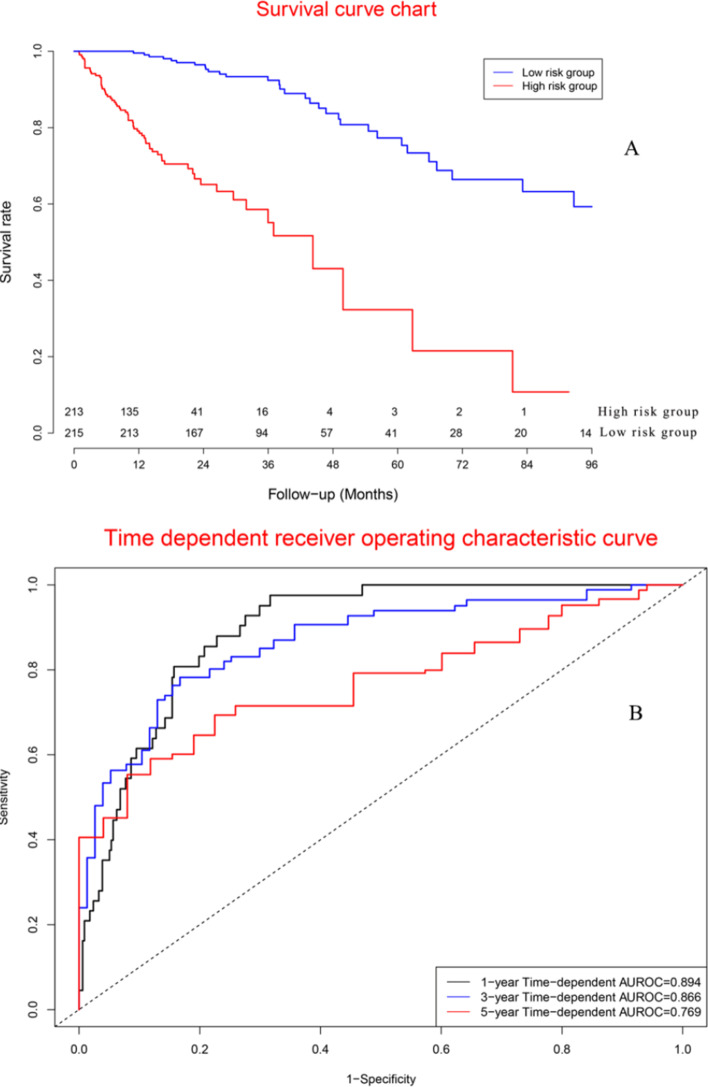
Clinical performance in validation cohort: **a** Survival curves for high risk group and low risk group; **b** Time-dependent receiver operation characteristic curves

Survival curves (Fig. 7A) demonstrated the mortality of high risk group was significantly poorer than that of low-risk group. Concordance indexes were 0.894, 0.866, and 0.769 for 1-year, 3-year, and 5-year survival (Fig. 7B). Additional file 5: Fig. S7 showed calibration curves of validation cohort.

### Correlation analyses

**Fig. 8:**
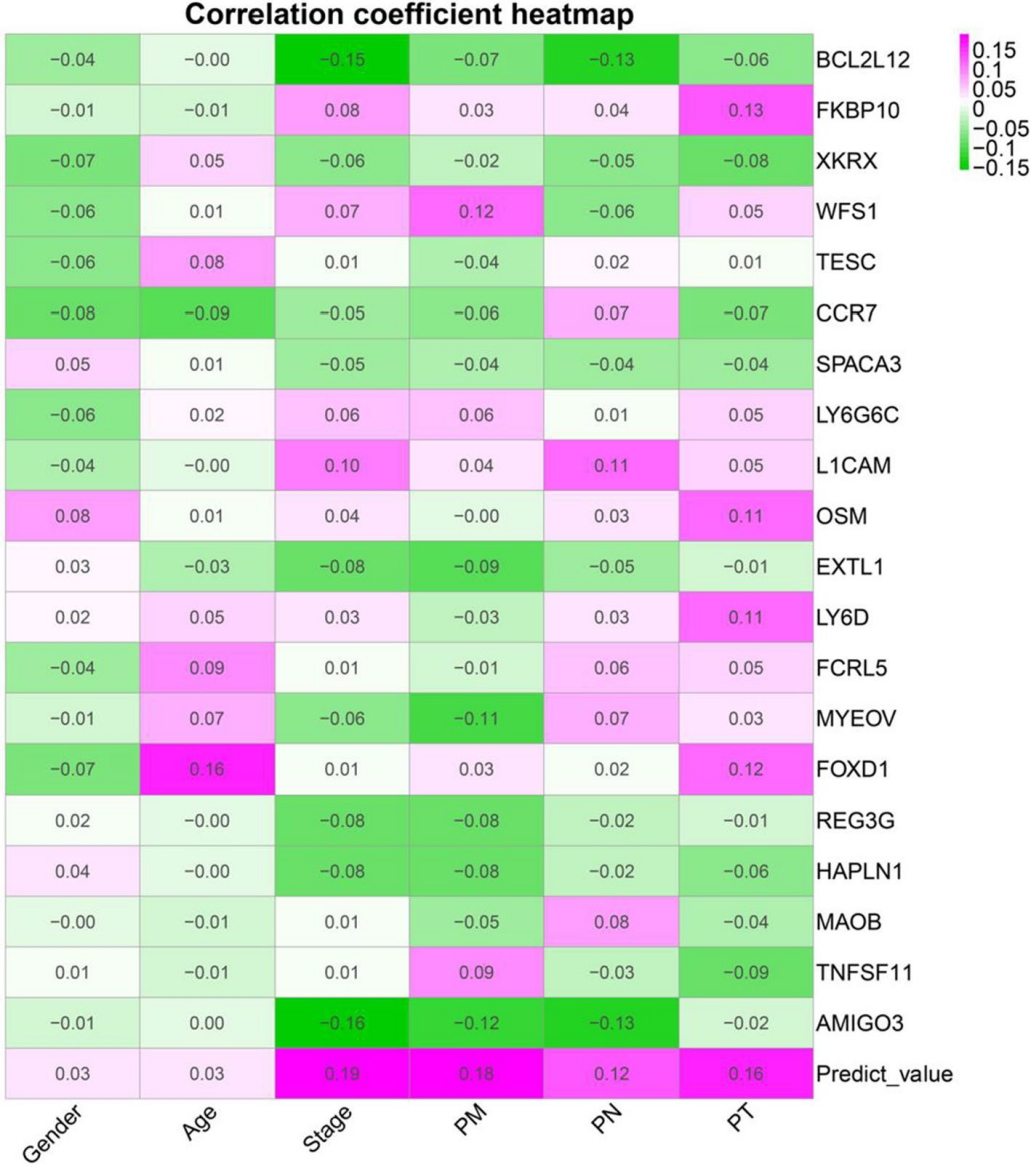
Correlation coefficient heatmap between immune genes and clinical variables

Correlation analyses (Fig. 8) showed prognostic score was positively correlated with pathological stage, the American Joint Committee on Cancer (AJCC) PM, AJCC PT, and AJCC PT. Additional file 5: Fig. S8 presented correlation significance between clinical variables and immune genes.

### Independence assessment

Prognostic model, AJCC PM, and age were independent risk factors for OS in model cohort (Table 3). In validation cohort, prognostic model, AJCC PM, AJCC PT, and age were ascertained to be independent risk factors for OS.

**Table 3:** Results of cox regression analyses

Variable	Univariate analysis		*P* value	Multivariate analysis			*P* value
	HR	95% CI		Coefficient	HR	95% CI	
Model cohort (n = 556)							
Gender (male/female)	1.323	0.987–1.774	0.061	0.276	1.318	0.976–1.779	0.071
Age (high/low)	1.023	1.011–1.036	< 0.001	0.031	1.032	1.019–1.045	< 0.001
Stage (3–4/1–2)	1.824	1.364–2.439	< 0.001	0.457	1.580	0.877–2.846	0.128
AJCC PM (1–2/0)	4.851	3.389–6.945	< 0.001	1.353	3.870	2.567–5.832	< 0.001
AJCC PN (1–4/0)	1.456	1.091–1.943	0.011	− 0.244	0.784	0.454–1.352	0.381
AJCC PT (3–4/1–2)	2.036	1.042–3.979	0.037	0.457	1.579	0.805–3.100	0.184
Prognostic model (high/low)	4.921	3.472–6.974	< 0.001	1.467	4.337	3.046–6.174	< 0.001
Validation cohort (n = 428)							
Gender (male/female)	1.125	0.755–1.678	0.562	− 0.133	0.875	0.583–1.313	0.520
Age (high/low)	1.018	1.002–1.035	0.031	0.034	1.035	1.018–1.052	< 0.001
Stage (3–4/1–2)	2.780	1.842–4.197	< 0.001	0.542	1.719	0.531–5.565	0.366
AJCC PM (1–2/0)	3.073	2.057–4.593	< 0.001	0.773	2.166	1.403–3.343	< 0.001
AJCC PN (1–4/0)	2.688	1.785–4.047	< 0.001	0.448	1.565	0.511–4.799	0.433
AJCC PT (3–4/1–2)	3.299	1.442–7.550	0.005	0.949	2.582	1.082–6.163	0.033
Prognostic model (high/low)	6.787	4.212–10.940	< 0.001	2.092	8.101	4.945–13.270	< 0.001

The median of Prognostic model scores was used as the cut-off value to stratify gastric cancer patients into high risk group and low risk group

AJCC, the American Joint Committee on Cancer; HR, hazard ratio; CI, confidence interval

### Subgroup analyses

**Fig. 9:**
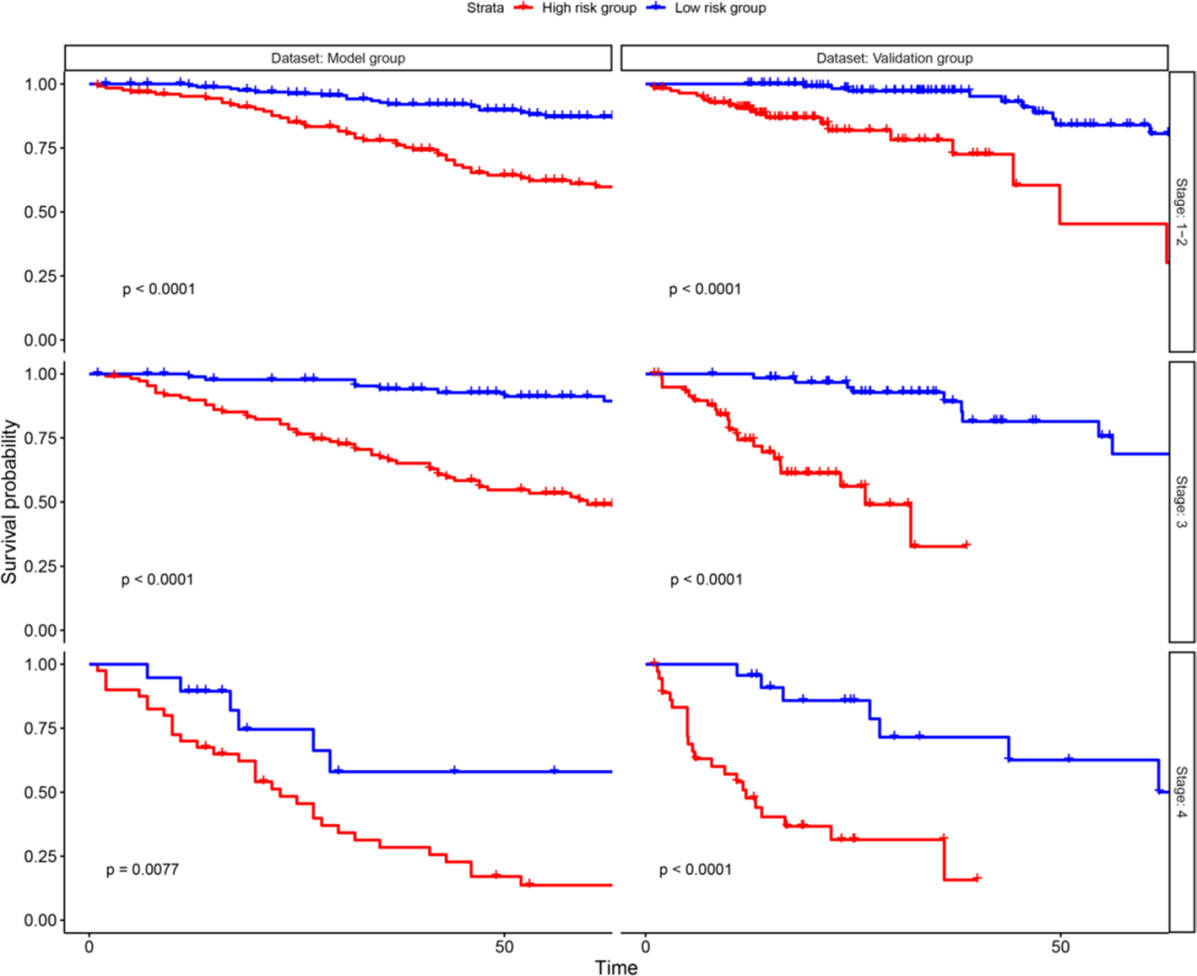
Subgroup survival analysis curve chart

Subgroup analyses were performed to explore the discriminate ability of prognostic model in different pathological stages. The results showed that the prognostic model has reliable discriminative ability in all pathological stages for model group and validation group (Fig. 9).

### Random survival forest model

Random survival forest (RFS) model was build for predicting OS based on previous immune genes. Random survival forest error rate chart (Additional file 5: Fig. S9) indicated that the model error rate dynamic changes according to different tree numbers. The predictive performance of RFS model was summarized in Additional file 5: Fig. S10.

Survival curves (Additional file 5: Fig. S11A) demonstrated the mortality of high risk group was significantly higher than that of low-risk group. Concordance indexes were 0.890, 0.869, and 0.899 for 1-year, 3-year, and 5-year survival (Additional file 5: Fig. S11B). Additional file 5: Fig. S12 showed calibration curves of RFS model.

### Multi-task logistic regression model

We further constructed Multi-task logistic regression (MTLR) model to predict OS for CRC patients. Survival curves (Additional file 5: Fig. S13A) demonstrated the mortality of high risk group was significantly higher than that of low-risk group. Concordance indexes were 0.841, 0.780, and 0.826 for 1-year, 3-year, and 5-year survival (Additional file 5: Fig. S13B). Additional file 5: Fig. S15 showed calibration curves of MTLR model.

### Comparisons of three prognostic models

**Fig. 10:**
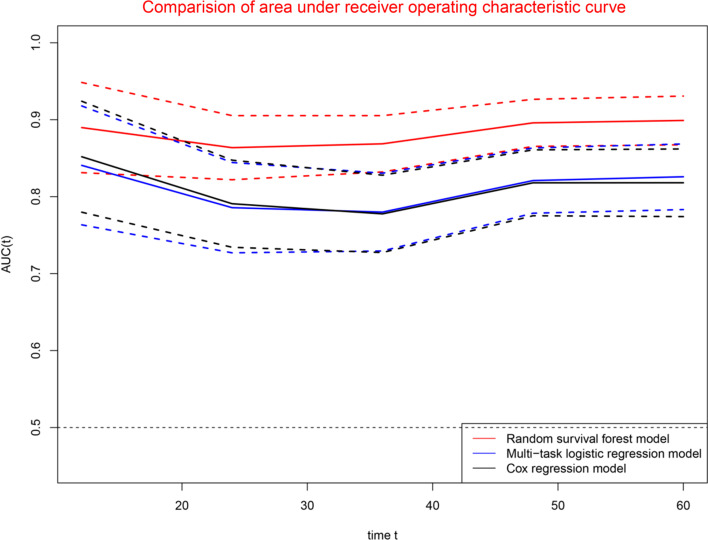
Predictive performance of three prognostic models. *Note*: The solid line represents the AUROC value, and the dash line represents the 95% confidence interval of the AUROC value

**Fig. 11:**
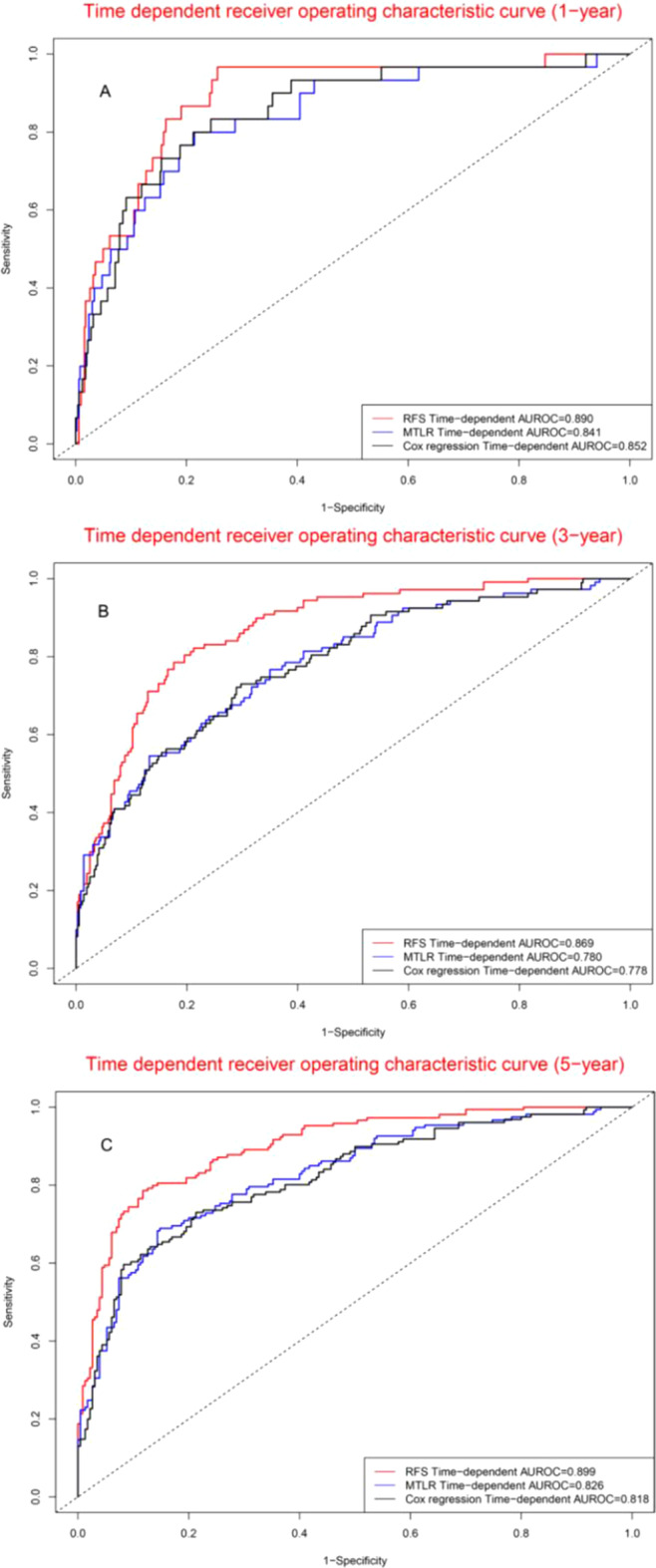
Comparison of areas under receiver operating characteristic curves: 1-year (**a**), 3-year (**b**) and 5-year (**c**)

Figure 10 demonstrated the dynamic changes of areas under the receiver operating characteristic curves for three prognostic models, suggesting that RFS model was superior to MTLR model and Cox model (The solid line represents the AUROC value, and the dash line represents the 95% confidence interval of the AUROC value in Fig. 10). Time dependent ROC curve analyses suggested that concordance index of RFS model was superior to that of MTLR model and Cox model for 1-year, 3-year, and 5-year survival (Fig. 11). The further comparisons demonstrated that the concordance index of RFS model was superior to that of Cox model except for 12 months, whereas concordance index of RFS model was superior to that of MTLR model for all time points (Table 4). The Brier score of RFS model, MTLR model, and Cox model were 0.144, 0.208, and 0.150, indicating diagnostic accuracy of RFS model was superior to that of MTLR model and Cox mode.

**Table 4:** Comparison of areas under receiver operating characteristic curves

Time point	AUROC			Paired comparison		
	RFS	MTLR	Cox	RFS versus MTLR	RFS versus Cox	MTLR versus Cox
12	0.890	0.841	0.852	0.027	0.055	0.236
24	0.864	0.786	0.791	0.001	0.001	0.847
36	0.869	0.780	0.778	0.001	0.001	0.983
48	0.896	0.821	0.818	0.001	0.001	0.942
60	0.899	0.826	0.818	0.001	0.001	0.370

AUROC, areas under receiver operating characteristic curves; RFS, random survival forest; MTLR, multi-task logistic regression

### Machine learning survival predictive system

**Fig. 12:**
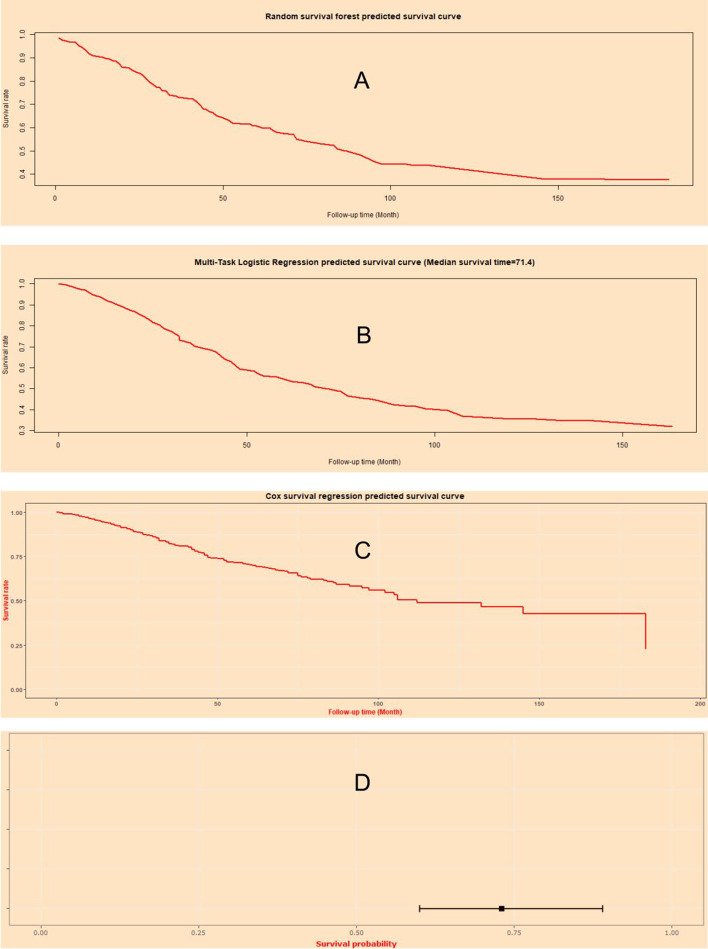
Home page of artificial intelligence survival prediction. **A** Predictive personal survival curve by random survival forest. **B** Predictive personal survival curve by multi-task logistic. **C** Predictive personal survival curve by Cox survival regression. **D** Mortality rate and 95% confidence interval by Cox survival regression

Machine learning survival predictive system was constructed for individual mortality risk prediction for CRC patients (Fig. 12), which was available at: https://zhangzhiqiao8.shinyapps.io/Artificial_Intelligence_Survival_Prediction_for_CRC_B1005_1/.

Machine learning survival predictive system provided individualized mortality risk predictive curve based on three machine learning algorithms: RFS model (Fig. 12A), MTLR model (Fig. 12B), and Cox model (Fig. 12C). Additionally, MTLR algorithm further provided median survival time in Fig. 12B. Cox survival regression algorithm provided predicted mortality percentage and 95% confidence interval for selected time points in Fig. 12D.

### Gene survival analysis screen system

Gene Survival Analysis Screen System was constructed for exploratory research of immune genes (Additional file 5: Fig. S15), which was available at: https://zhangzhiqiao8.shinyapps.io/Gene_Survival_Subgroup_Analysis_18_CRC_B1005/.

### Shapley additive instruction

**Fig. 13:**
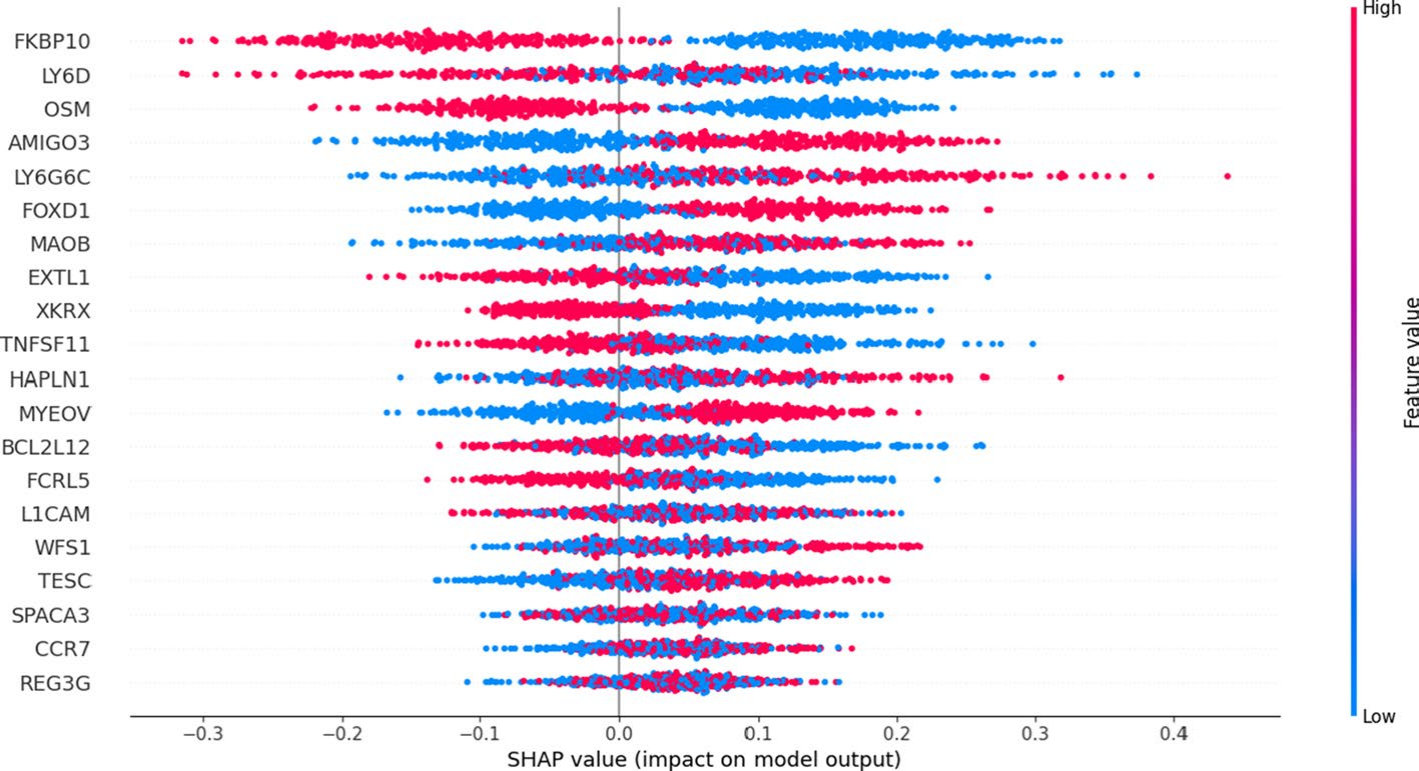
Shapley additive instruction distribution chart of included genes

Shapley additive instruction (SHAP) is a method that can be used to interpret the output of machine learning models. In order to show the importance of included prognostic genes in the prognostic model and its effect on prognosis, we drew the SHAP values of 20 included prognostic genes for each patient. The SHAP value distribution chart of different genes showed the direction and degree of the influence of each prognostic gene on the output of the model (Fig. 13). Each point in the Fig. 13 represents an individual patient. Red represents a high SHAP value, and blue represents a lower SHAP value.

## Discussion

The current study identified twenty immune genes as prognostic markers for overall survival of colorectal cancer. Through protein–protein interaction regulatory network, the current research described potential regulatory relationships among immune genes and transcription factors. Through three machine learning algorithms, the current research established an individual mortality risk predictive system for CRC patients. Based on individual mortality risk curves predicted by three machine learning algorithms, our machine learning survival predictive system could accurately predict the individual mortality risk of CRC patients.

The previous prognostic models provided predicted mortality percentages for different subgroups, but not the individual mortality risk curve for a special patient [23, 24]. Based on different machine learning algorithms, the current study provided three individual mortality risk predictive curves. The results of three individual mortality risk predictive curves were similar to a certain extent, providing a reliable individual mortality risk predictive method for CRC patients. Meanwhile, the current study further provided median survival time, predicted mortality percentage, and 95% confidence interval, which were superior to previous prognostic models.

As a non-parametric algorithm for Time-to-event data, random survival forest was regarded as a better method for prognostic prediction and variable selection [50, 51]. Random survival forest could solve the multicollinearity problem and was suitable for high dimensional survival data [52]. Because of high flexibility and non-parametric characteristics, random survival forest has been used for biomedical high dimensional survival data [53, 54]. The predictive accuracy of RSF model was superior to that of Cox model in cardiac arrhythmias patients [52]. Similar to the previous study [52], concordance indexes and Brier score suggested that the predictive accuracy of RFS model was superior to that of Cox model in current study. To date, there were few researches on MTLR model for prognostic studied.

Biological processes of immune genes were determined through TISIDB database. Major biological processes of tumor necrosis factor (ligand) superfamily, member 11 (TNFSF11) were leukocyte differentiation, acute inflammatory response, and regulation of leukocyte activation. Major biological processes of regenerating islet-derived 3 gamma (REG3G) were activation of innate immune response, toll-like receptor signaling pathway, and acute inflammatory response. Major biological processes of lymphocyte antigen 6 complex, locus D (LY6D) were leukocyte differentiation, lymphocyte differentiation, and response to stilbenoid. Major biological processes of sperm acrosome associated 3 (SPACA3) were response to virus, phagocytosis, and regulation of leukocyte activation. Major biological processes of chemokine (C–C motif) receptor 7 (CCR7) were dendritic cell chemotaxis, dendritic cell antigen processing and presentation, and establishment of T cell polarity. Major biological processes of BCL2-like 12 (BCL2L12) were aging, negative regulation of peptidase activity, and negative regulation of proteolysis. Major biological processes of FK506 binding protein 10 (FKBP10) were protein peptidyl-prolyl isomerization, protein folding, and peptidyl-proline modification. Major biological processes of tescalcin (TESC) were negative regulation of protein kinase activity, leukocyte differentiation, and protein targeting to membrane. Major biological processes of L1 cell adhesion molecule (L1CAM) were axonogenesis, positive regulation of cell growth, and regulation of cell size. Major biological processes of oncostatin M (OSM) were acute inflammatory response, positive regulation of defense response, and positive regulation of response to external stimulus.

The prognosis of BCL2L12 negative colon cancer patients was significantly poorer than that of BCL2L12 positive colon cancer patients [55]. High CCR7 positive cell density was significantly related to prognosis in colorectal cancer [56]. Colorectal cancer patients with high expression of L1CAM have higher risk of early metastasis [57]. FKBP10 might play an important role in the development of gastric cancer through cell adhesion molecules and extracellular matrix receptors [58]. High expression of HAPLN1 could upregulate the tumorigenicity of mesothelioma [59]. OSM was negative correlated with poor survival in breast cancer patients [60]. LY6D immunoreactivity was related to the invasiveness of ER positive breast cancer patients [61]. MYEOV stimulated the migration of colorectal cancer cells and promoted the proliferation and invasion of colorectal cancer [62]. FOXD1 promoted the progression of colorectal cancer through ERK 1/2 pathway [63].

Previous study suggested that immune microenvironment was closely related to tumorigenesis [14, 64]. F nucleus might inhibit anti-tumor immune response by reducing the density of CD4+ T cells in colorectal cancer [65]. PD-L1 promoted the development of colon cancer by reducing the antitumor immunity of CD8+ T cells [66]. FOXM1 inhibited the maturation of dendritic cells in colorectal cancer [67]. There was a correlation between the activity of natural killer cells and the development of tumor [68]. There was a negative correlation between eosinophil count values and risk of colorectal cancer [69]. Macrophage migration inhibitory factor could regulate the development of colorectal cancer [70]. High mast cell density indicates good prognosis for colon cancer [71]. High expression of monocyte was related to the poor prognosis of CRC patients [72]. Neutrophil to lymphocyte ratio was related with prognosis of colorectal cancer patients [73].

The current research established an individual mortality risk predictive system for CRC patients with the following advantages: First, based on three machine learning algorithms, the current research provided three individual mortality risk predictive curves, which was valuable for individualized treatment decision before surgery. These three prognostic models provided strong support for each other's reliability. Second, the current Machine learning survival predictive system provided median survival time, predicted mortality percentage, and 95% confidence interval, which were important for improving individualized treatment decision.

Shortcomings: First, the mortality rates in model group and validation group were 22.9% and 33.6%, respectively. High censoring rates of study datasets might weaken the convincing power of accuracy evaluation of prognostic models to a certain extent. Second, as a prognostic model, the sample size of the current research was relatively small, which was not enough to provide a convincing conclusion for clinical application. Third, large sample size and high quality follow-up management are very important for tumor long-term prognostic study. However, independent external verification cohorts often require a large sample size, long-term follow-up management and a large amount of research funding. It is very difficult for small research teams to set up a private independent external validation cohort. Therefore we selected external verification cohort (from GEO database) as external validation cohort. Fourth, several important variables, including information of radiotherapy, chemotherapy, and biotherapy, were not included in the current analysis. Fifth, GSE39582 dataset lacks some important basic information such as lymphovascular invasion, vascular invasion, residual tumor, and perineural invasion, affecting the general judgment of the model to a certain extent. Prospective, multicenter, and large sample size clinical studies are helpful to verify the clinical application value of the current prognostic model. Sixth, The tumor samples (n = 480) and normal samples (n = 41) are highly imbalanced in TCGA cohort for differentially expressed analyses. The sample imbalance may affect the results of differential expression analysis to some extent, thus affecting the differentially expressed genes. Considering the problem of sample imbalance, the differentially expressed genes in the current study need to be confirmed by larger sample size and more balanced data set.

## Conclusion

In conclusion, the current study identified twenty prognostic immune genes for CRC patients and constructed an immune-related regulatory network. Based on three machine learning algorithms, the current research provided three individual mortality predictive curves. The Machine learning survival predictive system was available at: https://zhangzhiqiao8.shinyapps.io/Artificial_Intelligence_Survival_Prediction_for_CRC_B1005_1/, which was valuable for individualized treatment decision before surgery.

## Supplementary Information

**Additional file 1.** Program application manual.

**Additional file 2.** Gene enrichment analysis dataset.

**Additional file 3.** SHAP application example in python.

**Additional file 4.** Statistics analysis example in R language.

**Additional file 5.** Supplementary Figure 1-15 (fifteen figures in total).

**Additional file 6.** Original dataset for analysis.

## Data Availability

The study data is available at: https://zhangzhiqiao8.shinyapps.io/Gene_Survival_Subgroup_Analysis_18_CRC_B1005/.

## Abbreviations

CRC: Colorectal cancer
TCGA: The cancer genome atlas
GEO: The gene expression omnibus
ROC: Receiver operating characteristic
DFS: Disease free survival
HR: Hazard ratio
CI: Confidence interval
AJCC: The American Joint Committee on Cancer
SD: Standard deviation
MTLR: Multi-task logistic regression
RFS: Random survival forest
