# Supplementary material for: Bioinformatics analysis reveals immune prognostic markers for overall survival of colorectal cancer patients: a novel machine learning survival predictive system
Source: BMC Bioinformatics. 2022 Apr 8;23:124. doi: 10.1186/s12859-022-04657-3 (PMC8991575; doi:10.1186/s12859-022-04657-3)

Program application manual

The current study presented two web computing tools for clinical translational application.

The first web tool is Artificial intelligence survival prediction system for prognostic prediction. Artificial intelligence survival prediction system has the following advantages:

1. Novel prognostic model: Artificial intelligence survival prediction system tools were constructed on the following ideas: three artificial intelligence algorithms + noninvasive method (gene expression data) + nomogram chart + web tool + full-time individual mortality risk prediction. To the best of our knowledge, the Artificial intelligence survival prediction system is the first one to provide full-time individual mortality risk prediction through web calculator based on gene expression data.

The web calculator can be used by click the following URL: <https://zhangzhiqiao8.shinyapps.io/Artificial_Intelligence_Survival_Prediction_for_CRC_B1005_1/>

Enter gene expression data values in the main page and click the "predict" function button. Different prediction results can be obtained by clicking the corresponding function key in Third step.


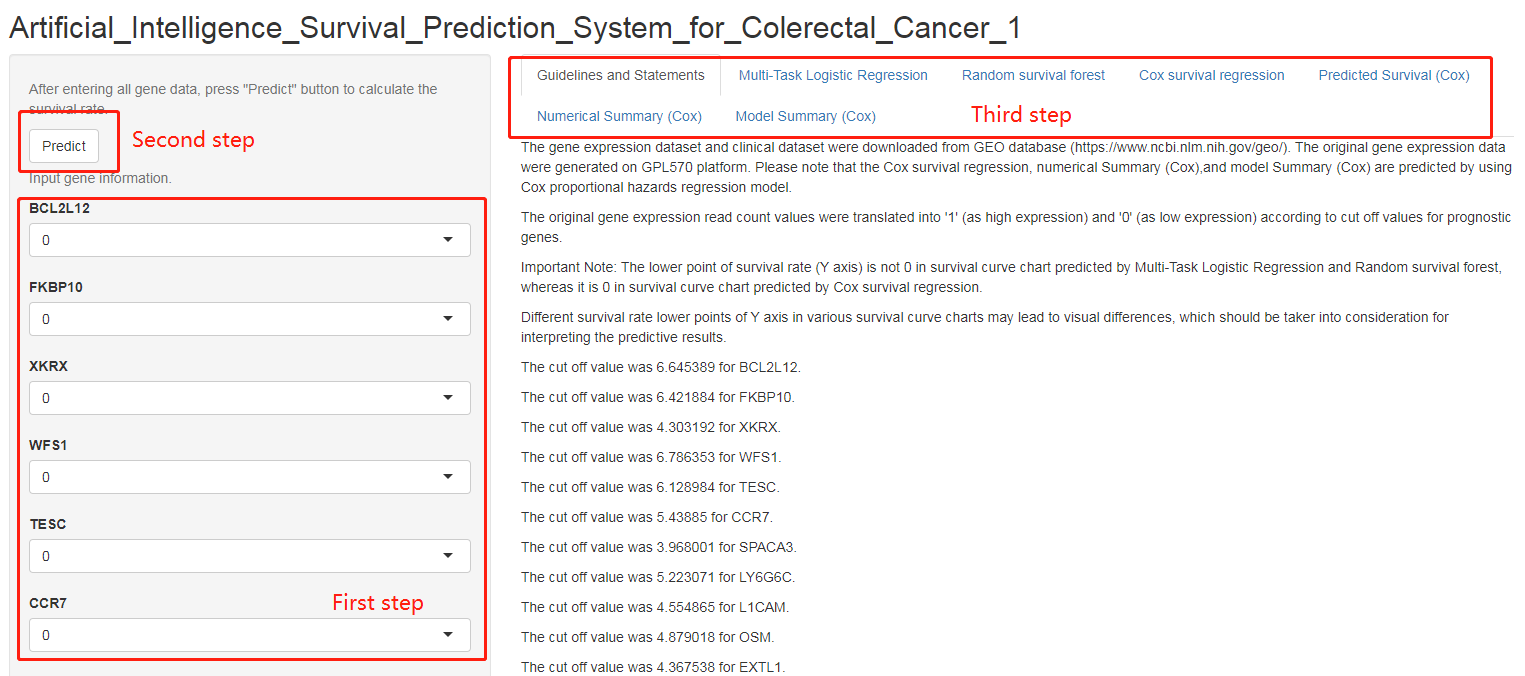


2. Full-time individual risk prediction: Artificial intelligence survival prediction system tool can provide full-time individual mortality risk prediction through an individual survival curve for a particular individual.


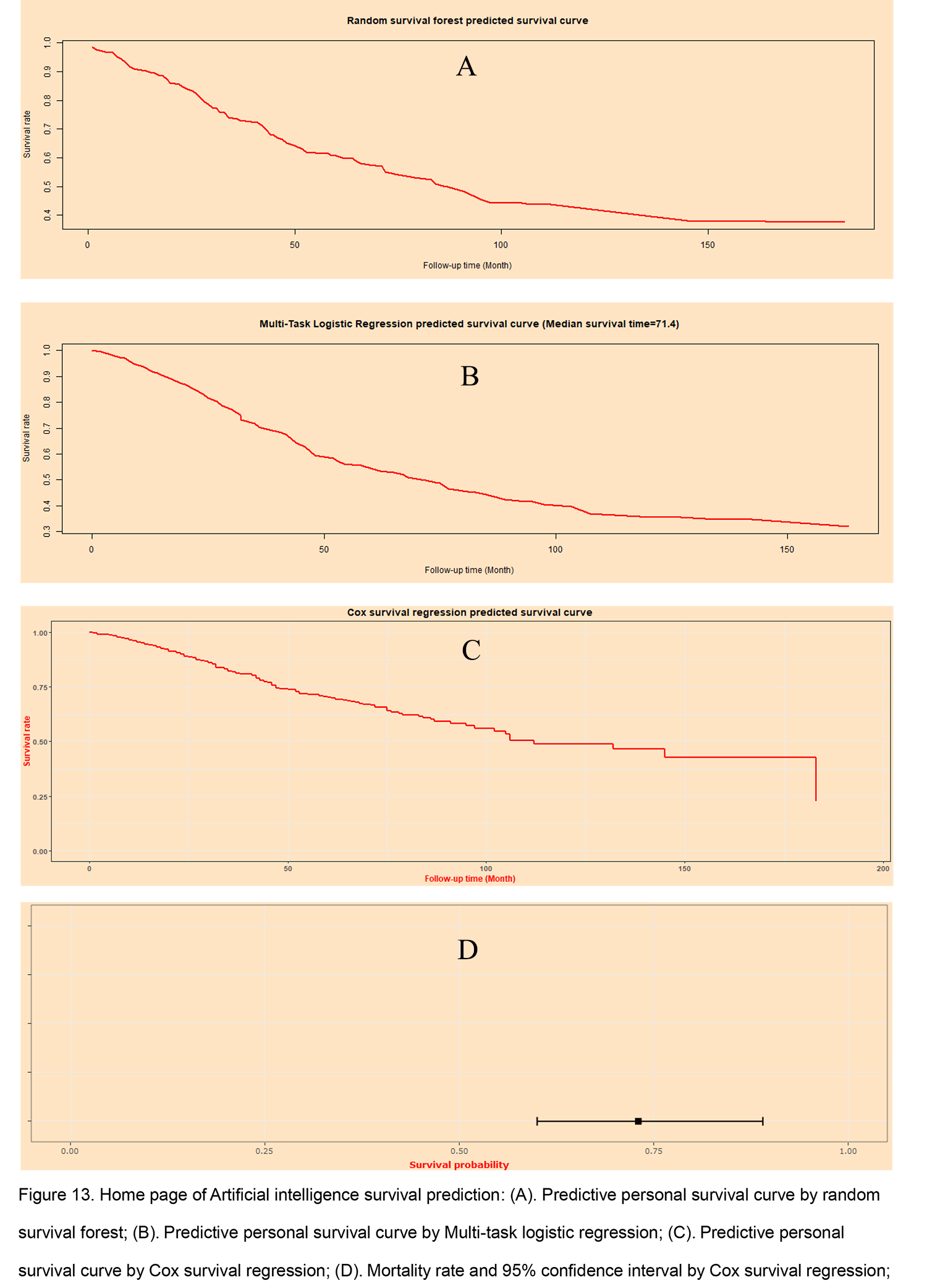


3. Artificial intelligence survival prediction system further provided median survival time, predicted mortality percentage, and 95% confidence interval, which were important for improving individualized treatment decision.

4. Visual illustration: Artificial intelligence survival prediction system tool can provide individual mortality risk prediction percentage and 95%CI according special time points (such as 12, 24, 36, 48, 60,and 72 months) selected by patients demonstrated by line graph.


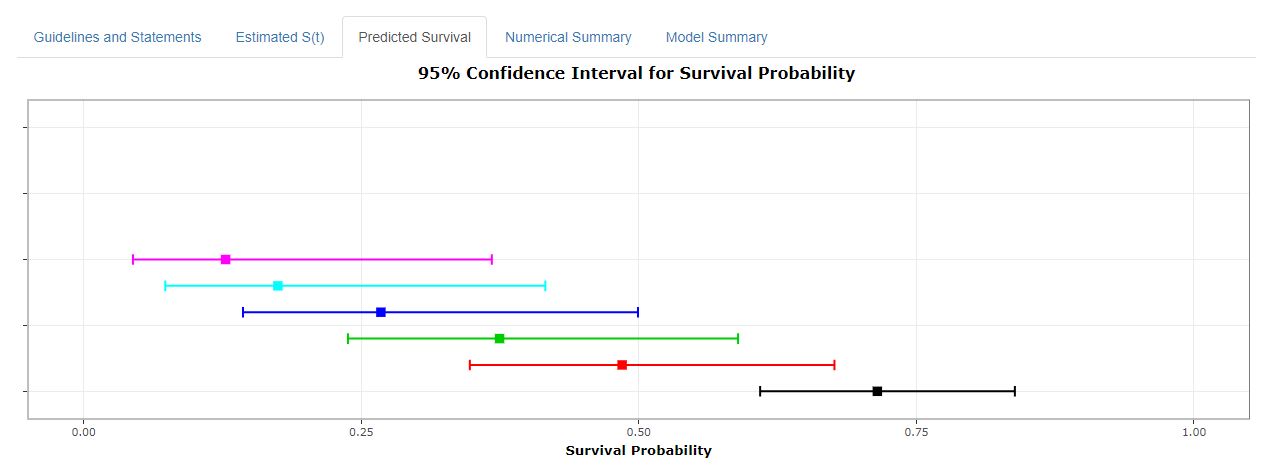


5. Numerical presentation: The results of individual mortality risk prediction percentage and 95%CI can be presented through simple table and special values.


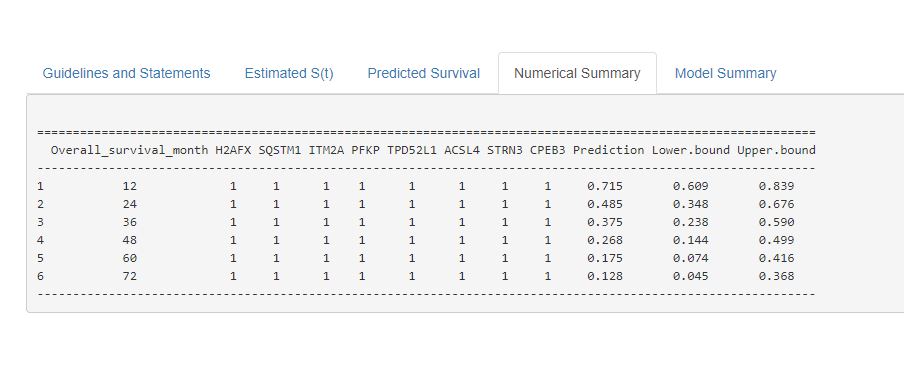


The second web tool is Gene Survival Analysis Screen System. Gene Survival Analysis Screen System is available at the following URL: https://zhangzhiqiao8.shinyapps.io/Gene_Survival_Subgroup_Analysis_18_CRC_B1005/.

This program has the following characteristics:

1. The program allowed users to download the dataset in current research and upload personal dataset for individual exploration.


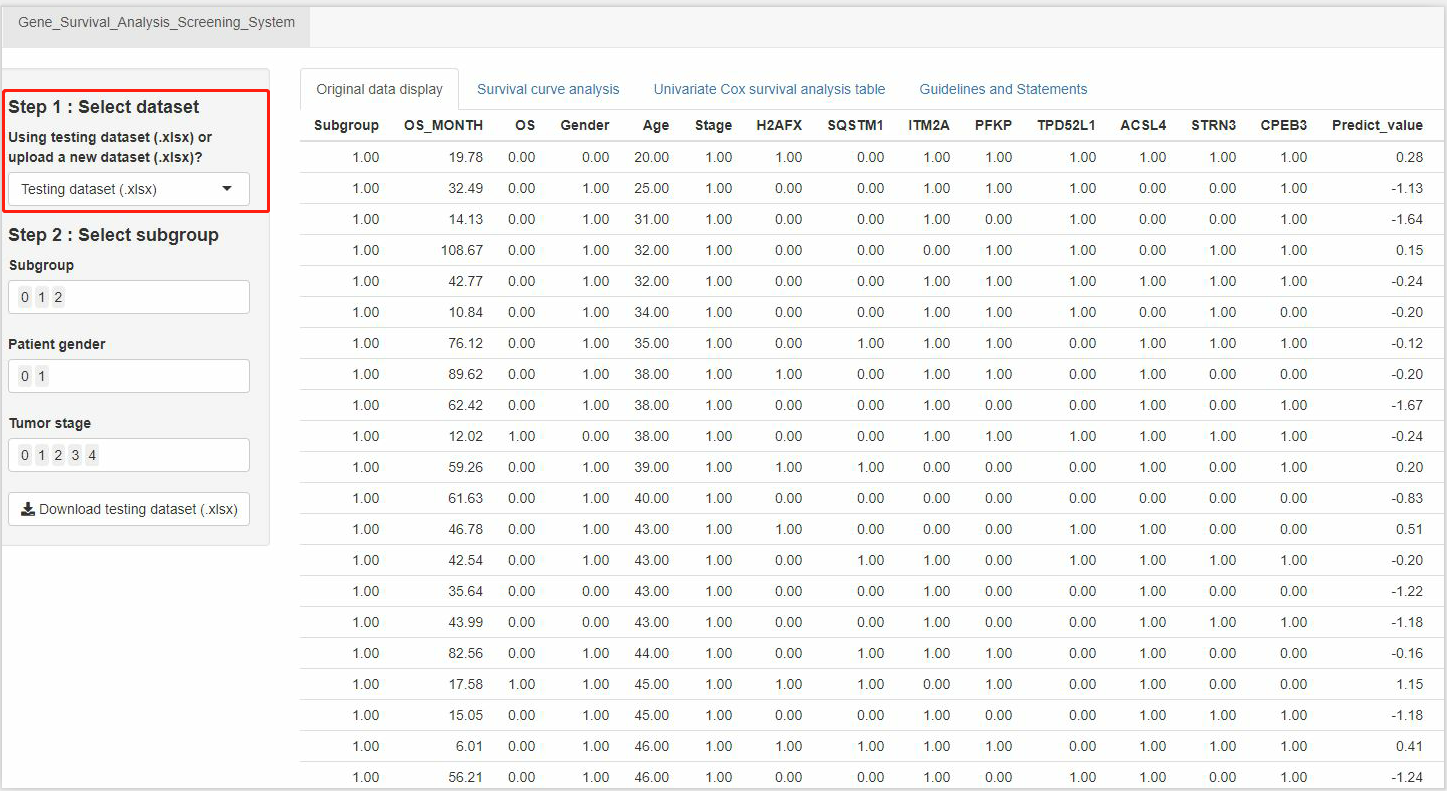


2. Users can define different gender and pathological stages as new study cohort according to their individual study purpose.


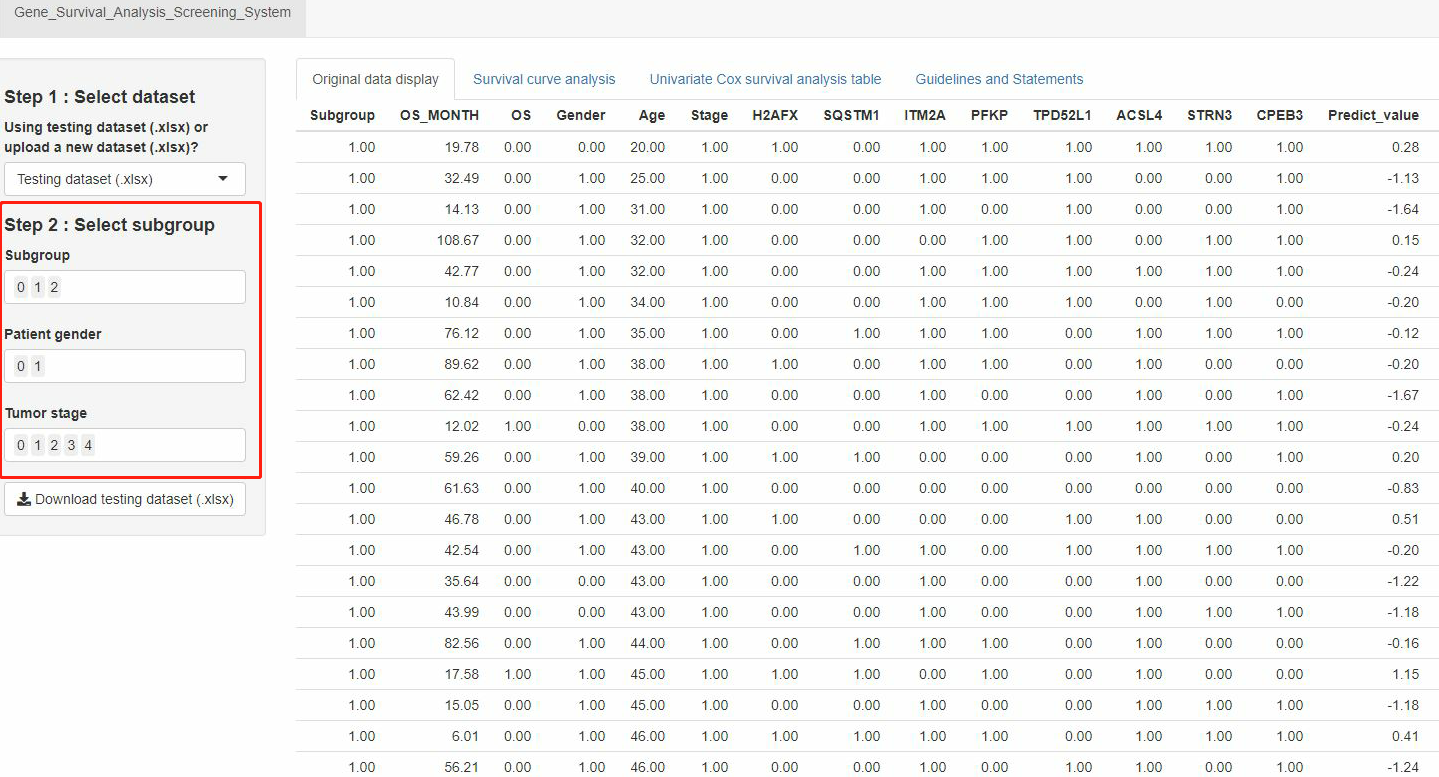


3. The results of survival analysis were presented as survival curve figure and survival analysis table, which were convenient for clinical application.

Survival curve figure


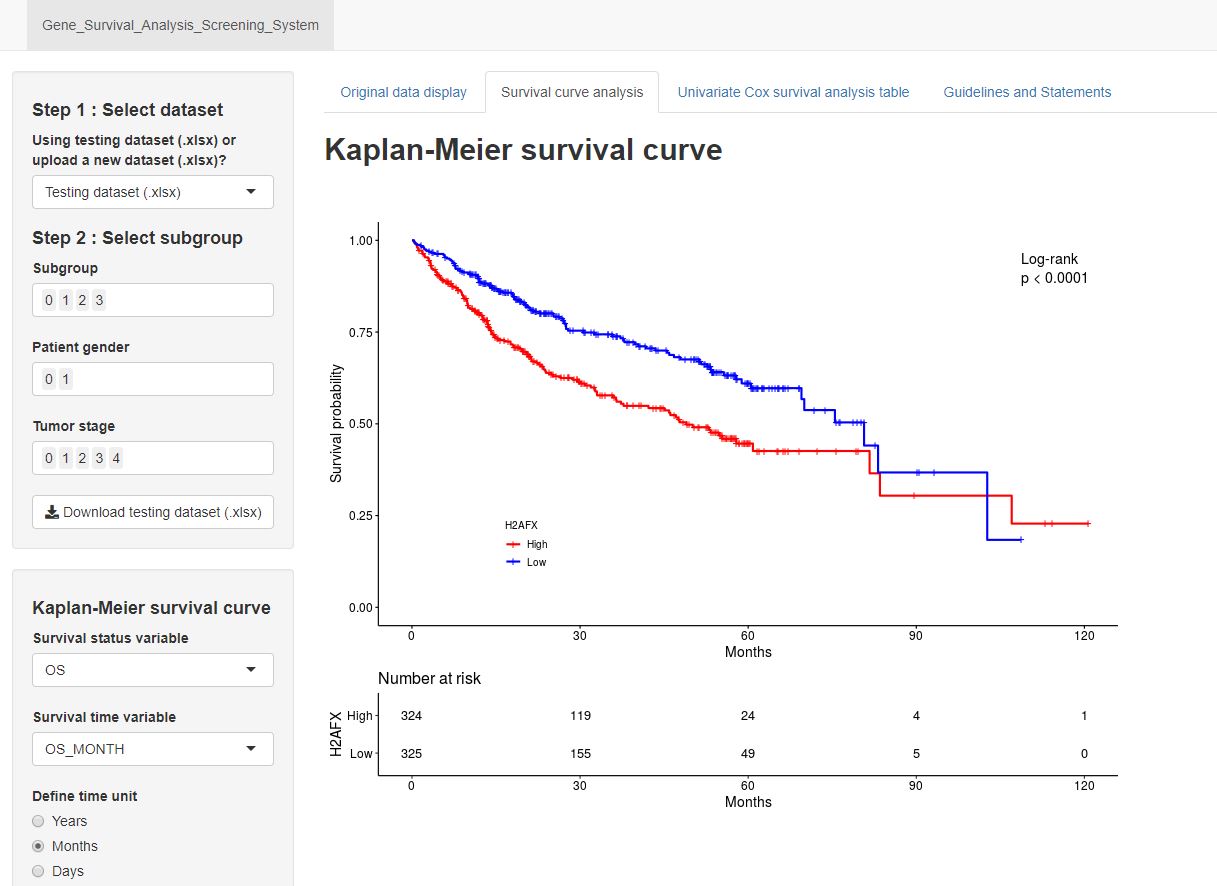


Survival analysis table


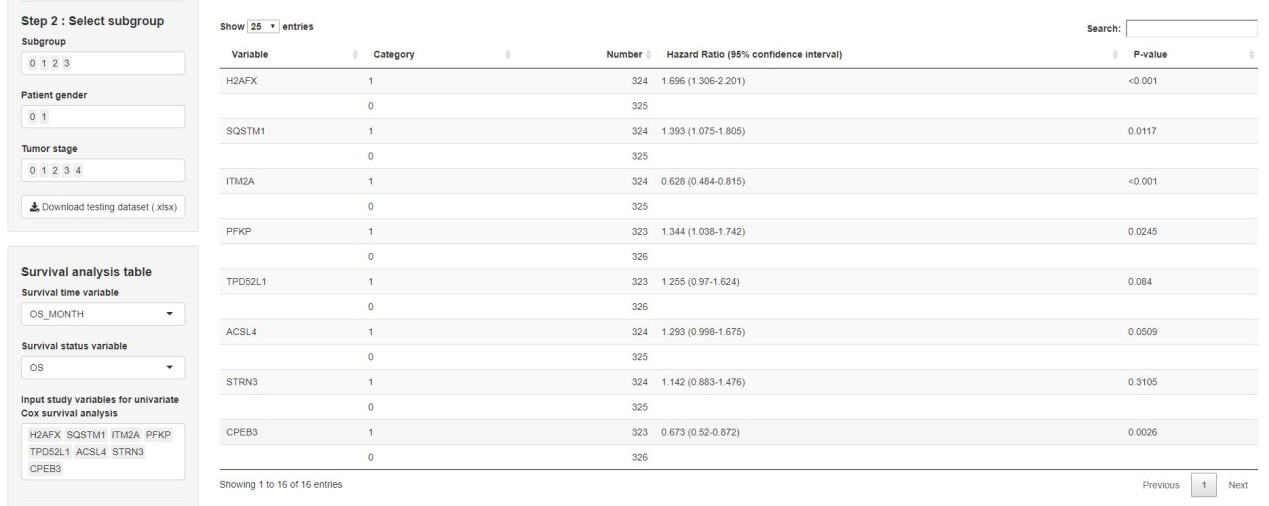

Supplement: Supplementary file 1 — Additional file 1. Program application manual. [file 12859_2022_4657_MOESM1_ESM.doc]
