# Supplementary material for: Bioinformatics analysis reveals immune prognostic markers for overall survival of colorectal cancer patients: a novel machine learning survival predictive system
Source: BMC Bioinformatics. 2022 Apr 8;23:124. doi: 10.1186/s12859-022-04657-3 (PMC8991575; doi:10.1186/s12859-022-04657-3)
Supplement: Supplementary file 4 — Additional file 4. Statistics analysis example in R language. [file 12859_2022_4657_MOESM4_ESM.r]

server <- function(input, output, session){

data_input <- reactive({

if(input$file2== 'Example2'){

d<- read.xlsx("model_dataset.xlsx",sheet=1,rowNames=FALSE,colNames = TRUE)

}

else if(input$file2 == 'load_my_own2'){

inFile <- input$file22

if (is.null(inFile))

return(NULL)

else if(grepl(".xlsx", inFile[1])) { d = read.xlsx(as.character(inFile$datapath), colNames = TRUE, rowNames = F) }

else if(grepl(".csv", inFile[1])) { d = read.csv(as.character(inFile$datapath), header = TRUE, sep = ",", stringsAsFactors = F, as.is = T, fill = T) }

else if(grepl(".txt", inFile[1])) { d = read.table(as.character(inFile$datapath), header = TRUE, sep = "\t", stringsAsFactors = F, as.is = T, fill = T) }

}

else

return(NULL)

# dim(data)

Dataset2 <- data.frame(d)

Dataset3 <-filter(Dataset2, Dataset2$Tumor_type %in% input$select15 & Dataset2$Stage %in% input$select16 & Dataset2$Gender %in% input$select17 )

return(as.data.frame(Dataset3))

})

output$Existing_dataset <- downloadHandler(

filename <- function() {

paste0("Testing_dataset", ".csv", sep='')

},

content <- function(file) {

ds <- data_input()

#write.xlsx2(ds, file, sheetName = "ds", row.names = FALSE)

write.csv(ds, file, row.names = F)

}

)

observe({

dsnames2 <- colnames(data_input())

cb_options2 <- dsnames2 updateSelectInput(session, "select21", label = "Study variable",

choices = c(cb_options2, "All Patients"),

selected = cb_options2[7])

updateSelectInput(session, "select22", label = "survival time variable",

choices = cb_options2,

selected=cb_options2[2])

updateSelectInput(session, "select23", label = "survival status variable",

choices = cb_options2,

selected =cb_options2[3] )

updateSelectInput(session, "select24", label = "survival time variable",

choices = cb_options2,

selected=cb_options2[2])

updateSelectInput(session, "select25", label = "survival status variable", choices = cb_options2,

selected =cb_options2[3] )

updateSelectInput(session, "show_vars26", label = "Input study variables for univariate Cox survival analysis",

choices = cb_options2,

selected=cb_options2[7])

}) output$downloadEx2 <- downloadHandler(

filename <- function() {

paste('Testing data', Sys.time(),'.csv', sep='')

},

content <- function(file) {

ds2 <- data_input()

write.csv(ds2, file, row.names = FALSE)

}

)

output$kmplot <- renderPlot({

data2 <- data_input()

if(!is.null(data2)){

for (j in 1:ncol(data2)) {

if (class(data2[, j]) %in% c("character"))

data2[,j] <- as.factor(data2[,j])

else data2[,j] = data2[, j]

}

if (input$select21 == "All Patients") {

time <- data2[,input$select22]

censor <- data2[,input$select23]

dat <- cbind.data.frame(time, censor)

fit <- surfit(surv(as.numeric(time), as.numeric(factor(censor))) ~ 1, data = dat)

# Drawing curves

xlabel <- c("Years", "Months", "Days")

b <- c(1, 12, 365)

#TF <- c(TRUE, FALSE)

if (nlevels(xvar) > 0) {

res <- ggsurvplot(fit, data = dat, font.main = 18,

font.x = 14,

font.y = 14, xlab = xlabel[as.numeric(input$time)], pval = T, pval.method = T,

pval.coord = c(max(time)*0.9, 0.9), pval.method.coord = c(max(time)*0.9, 0.95),

font.tickslab = 12,legend = c(0.2, 0.2), legend.title = input$select21,

palette = c("red", "blue"), risk.table = TRUE, risk.table.y.text.col = TRUE)#, fun = function(y) y*100)

return(res)

} else {

res <- ggsurvplot(fit, data = dat, font.main = 18,

font.x = 14,

font.y = 14, xlab = xlabel[as.numeric(input$time)], pval = T, pval.method = T,

pval.coord = c(max(time)*0.9, 0.9), pval.method.coord = c(max(time)*0.9, 0.95),

font.tickslab = 12,legend = c(0.2, 0.2), legend.title = input$select21,

palette = c("red", "blue"),

risk.table = TRUE, risk.table.y.text.col = FALSE)#, fun = function(y) y*100)

return(res)

}

} else {

xvar <- data2[, input$select21]

time <- data2[,input$select22]

censor <- data2[,input$select23]

dat <- cbind.data.frame(time, censor, xvar)

if (is.null(xvar) | is.null(time) | is.null(censor)) {

return(NULL)

} else if (nlevels(xvar) > 30) {

return(NULL)

} else if (class(xvar) %in% c("factor")) {

dat$Group <- as.factor(xvar)

fit <- surfit(surv(as.numeric(time), as.numeric(factor(censor))) ~ dat$Group, data = dat)

# Drawing curves

xlabel <- c("Years", "Months", "Days")

b <- c(1, 12, 365)

#TF <- c(TRUE, FALSE)

if (nlevels(xvar) > 0) {

res <- ggsurvplot(fit, data = dat, font.main = 18,

font.x = 14,

font.y = 14, xlab = xlabel[as.numeric(input$time)], pval = T, pval.method = T,

pval.coord = c(max(time)*0.9, 0.9), pval.method.coord = c(max(time)*0.9, 0.95),

font.tickslab = 12,legend = c(0.2, 0.2), legend.title = input$select21, legend.labs = levels(dat$Group),

palette = c("red", "blue"), risk.table = TRUE, risk.table.y.text.col = TRUE)#, fun = function(y) y*100)

return(res)

} else {

res <- ggsurvplot(fit, data = dat, font.main = 18,

font.x = 14,

font.y = 14, xlab = xlabel[as.numeric(input$time)], pval = T, pval.method = T,

pval.coord = c(max(time)*0.9, 0.9), pval.method.coord = c(max(time)*0.9, 0.95),

font.tickslab = 12,legend = c(0.2, 0.2), legend.title = input$select21, legend.labs = levels(dat$Group),

palette = c("red", "blue"),

risk.table = TRUE, risk.table.y.text.col = FALSE)#, fun = function(y) y*100)

return(res)

}

}else if (class(xvar) %in% c("integer","numeric") ) {

if (length(levels(factor(xvar)))<5) {

dat$Group <- ifelse(dat[, 'xvar'] < mean(dat[, 'xvar']), "Low", "High")

Group222 <- as.factor(dat$Group)

} else {perc <- as.numeric(as.integer(input$cutoff2))

dat$Group <- ifelse(dat[, 'xvar'] < quantile(dat[, 'xvar'], perc/100, na.rm= TRUE), "Low", "High")

Group222 <- as.factor(dat$Group)}

dat <- cbind.data.frame(time, censor, Group222 )

fit <- surfit(surv(as.numeric(time), as.numeric(factor(censor))) ~ Group222 , data = dat)

# Drawing curves

xlabel <- c("Years", "Months", "Days")

b <- c(1, 12, 365)

#TF <- c(TRUE, FALSE)

if (nlevels(xvar) > 0) {

res <- ggsurvplot(fit, data = dat, font.main = 18,

font.x = 14,

font.y = 14, xlab = xlabel[as.numeric(input$time)], pval = T, pval.method = T,

pval.coord = c(max(time)*0.9, 0.9), pval.method.coord = c(max(time)*0.9, 0.95),

font.tickslab = 12,legend = c(0.2, 0.2), legend.title = input$select21, legend.labs = levels(dat$Group),

palette = c("red", "blue"), risk.table = TRUE, risk.table.y.text.col = TRUE)#, fun = function(y) y*100, type="cairo")

return(res)

} else {

res <- ggsurvplot(fit, data = dat, font.main = 18,

font.x = 14,

font.y = 14, xlab = xlabel[as.numeric(input$time)], pval = T, pval.method = T,

pval.coord = c(max(time)*0.9, 0.9), pval.method.coord = c(max(time)*0.9, 0.95),

font.tickslab = 12,legend = c(0.2, 0.2), legend.title = input$select21, legend.labs = levels(dat$Group),

palette = c("red", "blue"),

risk.table = TRUE, risk.table.y.text.col = FALSE)#, fun = function(y) y*100, type="cairo")

return(res)

}

} else {

return(NULL)

}

}

}

})

output$out2 <- renderDataTable({

data2 <- data_input()

for (j in 1:ncol(data2)) {

if (class(data2[, j]) %in% c("character","factor"))

data2[,j] <- as.factor(data2[,j])

else data2[,j] = data2[, j]

}

xvar <- data2[,input$show_vars26, drop = FALSE]

time <- data2[,input$select24]

censor <- data2[,input$select25]

options(warn=-1)

res <- list()

for (i in 1: ncol(xvar)){

x <- xvar[, i]

if (is.null(x) | is.null(time) | is.null(censor)) {

return(NULL)

} else if (nlevels(x) > 30) {

return(NULL)

}

else if (class(x) %in% c("integer", "numeric")){

if (class(x) %in% c("integer", "numeric")){

dat <- cbind.data.frame(time, censor, xvar=x)

if (length(levels(factor(dat[, 'xvar'])))<5) {

dat$Group <- as.factor(dat[, 'xvar'])

Group <- as.factor(dat$Group)

} else {perc <- as.numeric(as.integer(input$cutoff2))

dat$Group <- ifelse(dat[, 'xvar'] < quantile(dat[, 'xvar'], perc/100), "Low", "High")

Group <- as.factor(dat$Group)}

x <- Group

}

Variable <- c(colnames(xvar)[i], rep("", (length(levels(x))-1)))

#x <- C(x, contr.treatment, base=3)

fit <- coxph(surv(as.numeric(time), as.numeric(factor(censor))) ~ x)

temp <- cox.zph(fit)

assum.p.value <- ifelse(temp$table[3] < 0.001, "<0.001", paste0(round(temp$table[3], 3)))

assump <- c(assum.p.value, rep("", (length(levels(x))-1)))

sum <- summary(fit)

hazard.ratio = round(sum$conf.int[, 1], 3)

lower95 = round(sum$conf.int[, 3], 3)

upper95 = round(sum$conf.int[, 4], 3)

logrank.p.value = ifelse(sum$sctest[3] < 0.001, "<0.001", paste0(round(sum$sctest[3], 3)))

logrankp <- c(logrank.p.value, rep("", (length(levels(x))-1)))

type3.p.value = ifelse(sum$coefficients[, 5] < 0.001, "<0.001", paste0(round(sum$coefficients[, 5], 3)))

counts <- data.frame(table(x))

counts <- rbind.data.frame(counts[2:nrow(counts),], counts[1, ])

coxres <- cbind.data.frame(Variable, counts, c(paste0(hazard.ratio, " (", lower95,

"-", upper95, ")"), ""),logrankp)

colnames(coxres) <- c("Variable", "Category", "Number", "Hazard Ratio (95% confidence interval)", "P-value")

if (length(levels(factor(censor)))>0) {

res[[i]] <- coxres

} else if (length(levels(factor(censor)))<0) {

coxres2 <- cbind.data.frame(coxres, assump)

colnames(coxres2)[7] <- "P-value for Proportional Hazards Assumption"

res[[i]] <- coxres2

}

} else

return(NULL)

}

res_table <- do.call("rbind", res)

}, rownames = FALSE) #, options = list(dom = 'tip'))

output$pv23 <- renderTable({

head(data_input(),n=25)

})

output$pv21 <- renderUI({

hs3 <- paste("&emsp;")

hs4 <- paste("Kaplan-Meier survival curve")

HTML(paste(h2(strong(hs4)), hs3, sep = '<br/>'))

})

output$pv22 <- renderUI({

hs3 <- paste("&emsp;")

hs4 <- paste("Univariate survival analysis table")

HTML(paste(hs3, h2(strong(hs4)), hs3, sep = '<br/>'))

})

#output$out2 = renderUI ({

# tagList(

# htmlOutput("pv22"),

# dataTableOutput("out2")

# )

#})

output$downloadKM <- downloadHandler(

filename <- function() {

pdf_file <<- as.character(input$fname21)

paste('KM_', pdf_file, '.pdf', sep='')

},

content <- function(file) {

pdf(file=paste(pdf_file,".pdf",sep="") , height= 12, width=18)

#plot_fp()

data2 <- data_input()

for (j in 1:ncol(data2)) {

if (class(data2[, j]) %in% c("character"))

data2[,j] <- as.factor(data2[,j])

else data2[,j] = data2[, j]

}

if (input$select21 == "All Patients") {

time <- data2[,input$select22]

censor <- data2[,input$select23]

dat <- cbind.data.frame(time, censor)

fit <- surfit(surv(as.numeric(time), as.numeric(factor(censor))) ~ 1, data = dat)

# Drawing curves

xlabel <- c("Years", "Months", "Days")

b <- c(1, 12, 365)

#TF <- c(TRUE, FALSE)

if (nlevels(xvar) > 0) {

res <- ggsurvplot(fit, data = dat, font.main = 18,

font.x = 14,

font.y = 14, xlab = xlabel[as.numeric(input$time)], pval = T, pval.method = T,

pval.coord = c(max(time)*0.9, 0.9), pval.method.coord = c(max(time)*0.9, 0.95),

font.tickslab = 12,legend = c(0.2, 0.2), legend.title = input$select21,

palette = c("red", "blue"), risk.table = TRUE, risk.table.y.text.col = TRUE)#, fun = function(y) y*100)

p <- grid.arrange(res$plot, res$table, nrow = 2, heights=c(0.7,0.3))

plot(p)

} else {

res <- ggsurvplot(fit, data = dat, font.main = 18,

font.x = 14,

font.y = 14, xlab = xlabel[as.numeric(input$time)], pval = T, pval.method = T,

pval.coord = c(max(time)*0.9, 0.9), pval.method.coord = c(max(time)*0.9, 0.95),

font.tickslab = 12,legend = c(0.2, 0.2), legend.title = input$select21,

palette = c("red", "blue"),

risk.table = TRUE, risk.table.y.text.col = FALSE)#, fun = function(y) y*100)

p <- grid.arrange(res$plot, res$table, nrow = 2, heights=c(0.7,0.3))

plot(p)

}

} else {

xvar <- data2[, input$select21]

time <- data2[,input$select22]

censor <- data2[,input$select23]

dat <- cbind.data.frame(time, censor, xvar)

if (is.null(xvar) | is.null(time) | is.null(censor)) {

return(NULL)

} else if (nlevels(xvar) > 30) {

return(NULL)

} else if (class(xvar) == "factor") {

dat$Group <- as.factor(xvar)

fit <- surfit(surv(as.numeric(time), as.numeric(factor(censor))) ~ dat$Group , data = dat)

# Drawing curves

xlabel <- c("Years", "Months", "Days")

b <- c(1, 12, 365)

#TF <- c(TRUE, FALSE)

if (nlevels(xvar) > 0) {

res <- ggsurvplot(fit, data = dat, font.main = 18,

font.x = 14,

font.y = 14, xlab = xlabel[as.numeric(input$time)], pval = T, pval.method = T,

pval.coord = c(max(time)*0.9, 0.9), pval.method.coord = c(max(time)*0.9, 0.95),

font.tickslab = 12,legend = c(0.2, 0.2), legend.title = input$select21, legend.labs = levels(dat$Group),

palette = c("red", "blue"), risk.table = TRUE, risk.table.y.text.col = TRUE)#, fun = function(y) y*100)

p <- grid.arrange(res$plot, res$table, nrow = 2, heights=c(0.7,0.3))

plot(p)

} else {

res <- ggsurvplot(fit, data = dat, font.main = 18,

font.x = 14,

font.y = 14, xlab = xlabel[as.numeric(input$time)], pval = T, pval.method = T,

pval.coord = c(max(time)*0.9, 0.9), pval.method.coord = c(max(time)*0.9, 0.95),

font.tickslab = 12,legend = c(0.2, 0.2), legend.title = input$select21, legend.labs = levels(dat$Group),

palette = c("red", "blue"),

risk.table = TRUE, risk.table.y.text.col = FALSE)#, fun = function(y) y*100)

p <- grid.arrange(res$plot, res$table, nrow = 2, heights=c(0.7,0.3))

plot(p)

}

}else if (class(xvar) %in% c("integer", "numeric") ) {

if (length(levels(factor(dat[, 'xvar'])))<5) {

dat$Group <- ifelse(dat[, 'xvar'] < mean(dat[, 'xvar']), "Low", "High")

dat$Group <- as.factor(dat$Group)

} else {perc <- as.numeric(as.integer(input$cutoff2))

dat$Group <- ifelse(dat[, 'xvar'] < quantile(dat[, 'xvar'], perc/100), "Low", "High")

dat$Group <- as.factor(dat$Group)}

fit <- surfit(surv(as.numeric(time), as.numeric(factor(censor))) ~ dat$Group, data = dat)

# Drawing curves

xlabel <- c("Years", "Months", "Days")

b <- c(1, 12, 365)

#TF <- c(TRUE, FALSE)

if (nlevels(xvar) > 0) {

res <- ggsurvplot(fit, data = dat, font.main = 18,

font.x = 14,

font.y = 14, xlab = xlabel[as.numeric(input$time)], pval = T, pval.method = T,

pval.coord = c(max(time)*0.9, 0.9), pval.method.coord = c(max(time)*0.9, 0.95),

font.tickslab = 12,legend = c(0.2, 0.2), legend.title = input$select21, legend.labs = levels(dat$Group),

palette = c("red", "blue"), risk.table = TRUE, risk.table.y.text.col = TRUE)#, fun = function(y) y*100)

p <- grid.arrange(res$plot, res$table, nrow = 2, heights=c(0.7,0.3))

plot(p)

} else {

res <- ggsurvplot(fit, data = dat, font.main = 18,

font.x = 14,

font.y = 14, xlab = xlabel[as.numeric(input$time)], pval = T, pval.method = T,

pval.coord = c(max(time)*0.9, 0.9), pval.method.coord = c(max(time)*0.9, 0.95),

font.tickslab = 12,legend = c(0.2, 0.2), legend.title = input$select21, legend.labs = levels(dat$Group),

palette = c("red", "blue"),

risk.table = TRUE, risk.table.y.text.col = FALSE)#, fun = function(y) y*100)

p <- grid.arrange(res$plot, res$table, nrow = 2, heights=c(0.7,0.3))

plot(p)

}

} else {

return(NULL)

}

}

dev.off()

file.copy(paste(pdf_file,'.pdf', sep='') ,file, overwrite=TRUE)

})

output$downloadplot <- downloadHandler(

filename = "survival_analysis_curve_plot.png",

content = function(file) {

png(file) # open the png device

plot(x=x(), y=y(), main = "iris dataset plot", xlab = xl(), ylab = yl()) # draw the plot

dev.off() # turn the device off

}

)

output$x1 = downloadHandler(

filename <- function() {

csv_file <<- as.character(input$fname22)

#paste('survival_', csv_file,'.csv', sep='')

paste("Univariate_survival_analysis_result", ".csv", sep='')

},

content = function(file) {

data2 <- data_input()

for (j in 1:ncol(data2)) {

if (class(data2[, j]) %in% c("character","factor"))

data2[,j] <- as.factor(data2[,j])

else data2[,j] = data2[, j]

}

xvar <- data2[,input$show_vars26, drop = FALSE]

time <- data2[,input$select24]

censor <- data2[,input$select25]

options(warn=-1)

res <- list()

for (i in 1: ncol(xvar)){

x <- xvar[, i]

if (is.null(x) | is.null(time) | is.null(censor)) {

return(NULL)

} else if (nlevels(x) > 30) {

return(NULL)

} else if ( class(x) %in% c("integer", "numeric")){

if (class(x) %in% c("integer", "numeric")){

dat <- cbind.data.frame(time, censor, xvar=x)

if (length(levels(factor(x)))<5) {

Group121 <- ifelse(x < mean(x), "Low", "High")

Group <- as.factor(Group121)

} else {perc <- as.numeric(as.integer(input$cutoff2))}

Group121 <- ifelse(x < mean(x), "Low", "High")

Group <- as.factor(Group121)

x <- Group

}

Variable <- c(colnames(xvar)[i], rep("", (length(levels(x))-1)))

#x <- C(x, contr.treatment, base=3)

fit <- coxph(surv(as.numeric(time), as.numeric(factor(censor))) ~ x)

temp <- cox.zph(fit)

assum.p.value <- ifelse(temp$table[3] < 0.001, "<0.001", paste0(round(temp$table[3], 3)))

assump <- c(assum.p.value, rep("", (length(levels(x))-1)))

sum <- summary(fit)

hazard.ratio = round(sum$conf.int[, 1], 3)

lower95 = round(sum$conf.int[, 3], 3)

upper95 = round(sum$conf.int[, 4], 3)

logrank.p.value = ifelse(sum$sctest[3] < 0.001, "<0.001", paste0(round(sum$sctest[3], 3)))

logrankp <- c(logrank.p.value, rep("", (length(levels(x))-1)))

type3.p.value = ifelse(sum$coefficients[, 5] < 0.001, "<0.001", paste0(round(sum$coefficients[, 5], 3)))

counts <- data.frame(table(x))

counts <- rbind.data.frame(counts[2:nrow(counts),], counts[1, ])

coxres <- cbind.data.frame(Variable, counts, c(paste0(hazard.ratio, " (", lower95,

"-", upper95, ")"), ""), logrankp)

colnames(coxres) <- c("Variable", "Category", "Number", "Hazard Ratio (95% confidence interval)", "P-value")

if (length(levels(factor(censor)))>0) {

res[[i]] <- coxres

} else if (length(levels(factor(censor)))<0) {

coxres2 <- cbind.data.frame(coxres, assump)

colnames(coxres2)[7] <- "P-value for Proportional Hazards Assumption"

res[[i]] <- coxres2

}

} else

return(NULL)

}

res_table <- do.call("rbind", res)

write.csv(res_table, file, row.names = F)

#write.xlsx2(res_table, file, sheetName = "ds", row.names = FALSE)

})

output$ReadMe2 <- renderUI({

str0 <- paste("&emsp;")

str1 <- paste("DATA FORMAT")

str2 <- paste("&emsp; 1. Data should be input as a .txt or .xlsx or .csv file. The first row of the data file have information about the variables.")

str3 <- paste("&emsp; 2. The remaining lines contain measurements one line per each subject/sample, described in the format below.")

str4 <- paste("&emsp; 3. The first column of the file contains the survival time 'time' (column 1), survival status 'status' (column 2) followed by the other variables of interest.")

str6 <- paste("&emsp;&emsp; a) Column_1. This should contain the survival time, for the user's reference.")

str7 <- paste("&emsp;&emsp; b) Column_2. This should contain the survival status, input as 'censored' vs 'dead'.")

str8 <- paste("&emsp;&emsp; c) Remaining Columns. These columns should contain information with variables of interest, such as age, race, gender and patient id kept as reference." )

str9 <- paste("NOTE1: In this tab, you are able to carry out standard survival analysis. On the left side panel, you have the options to upload data; select variables for univariate

survival association analysis with cox proportional hazard model; meanwhile, select the variables for Kaplan Meier analysis; download the output table and plot accordingly.")

str10 <- paste("NOTE2: To carry out univariate survival association analysis, it is based on cox proportional hazards model with entering one variable in the model each time. Select the variables of interest to generate the output table.

You can choose the option to be 'Yes' if you want to test the proportional hazard assumption using Schoenfeld residuals test.")

str11 <- paste("NOTE3: To carry out Kaplan Meier Analysis, the variable of interest can be All Patients or a categorical variable. You also need to specify the time unit corresponding to the data in order for the plot to display correctly.")

HTML(paste(str0, strong(str9), str0,str0,str0,strong((str10)), str0,str0,str0,strong((str11)), str0, str0,h5(strong(str1)), str0, str2, str3, str4, str6, str7,str8, str0, sep = '<br/>'))

})

}
