# Supplementary figures and images for: Bioinformatics analysis reveals immune prognostic markers for overall survival of colorectal cancer patients: a novel machine learning survival predictive system
Source: BMC Bioinformatics. 2022 Apr 8;23:124. doi: 10.1186/s12859-022-04657-3 (PMC8991575; doi:10.1186/s12859-022-04657-3)

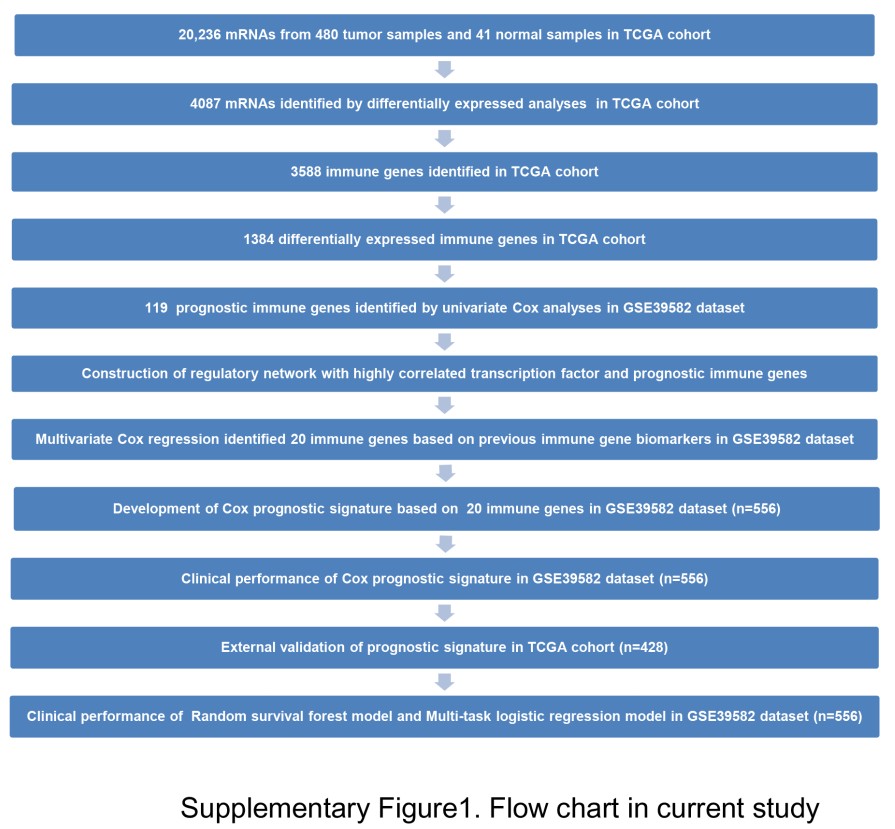


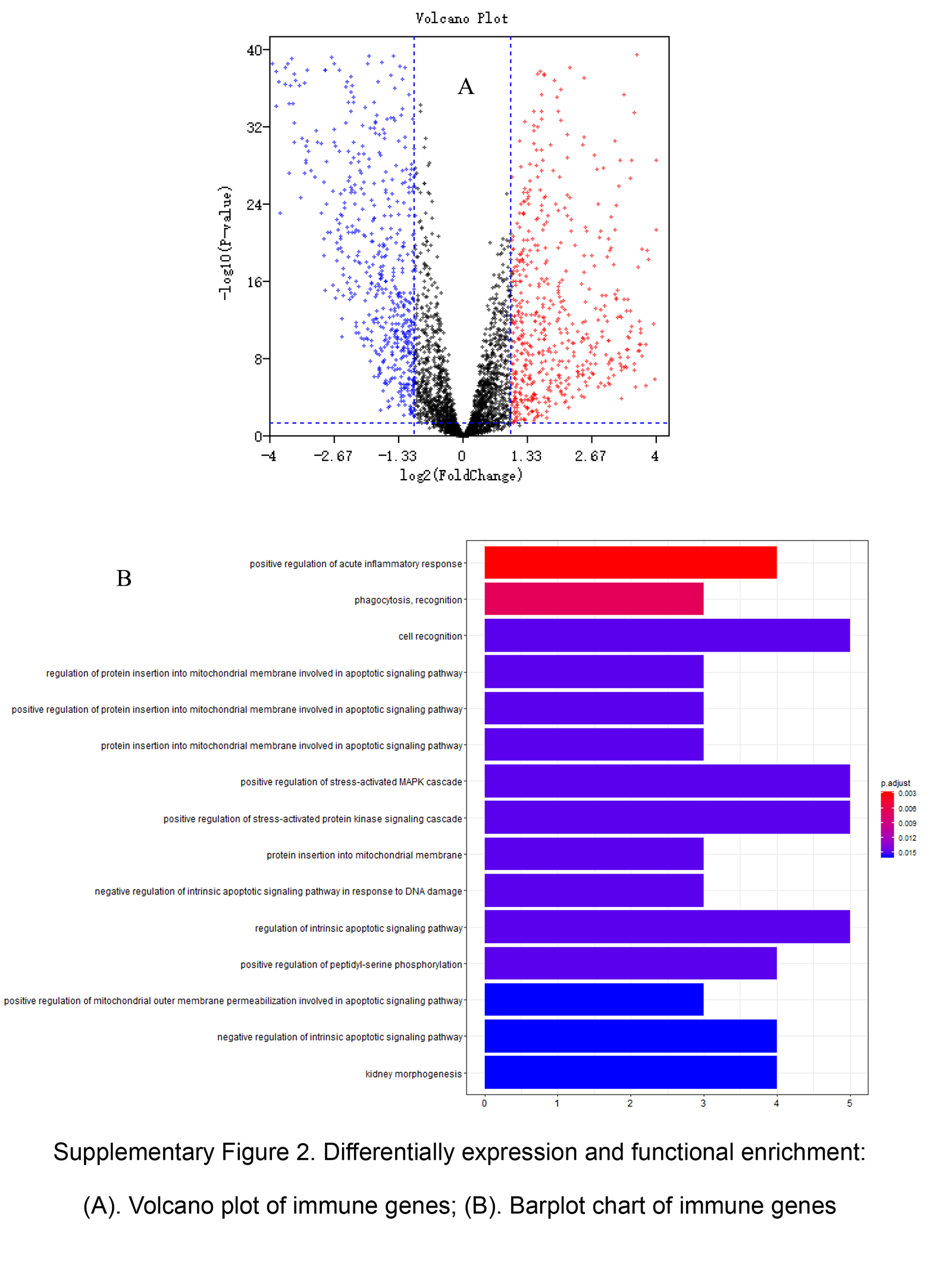


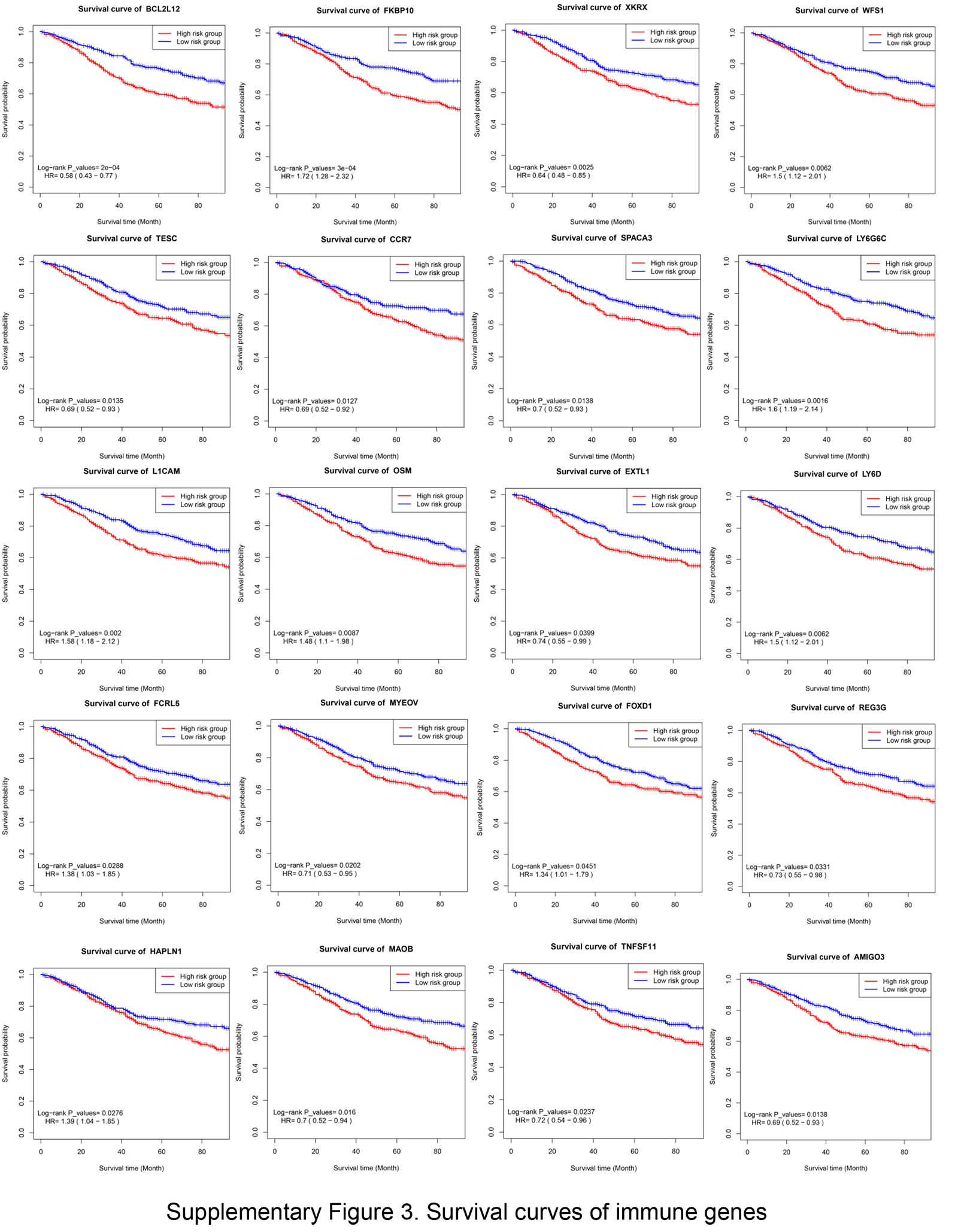


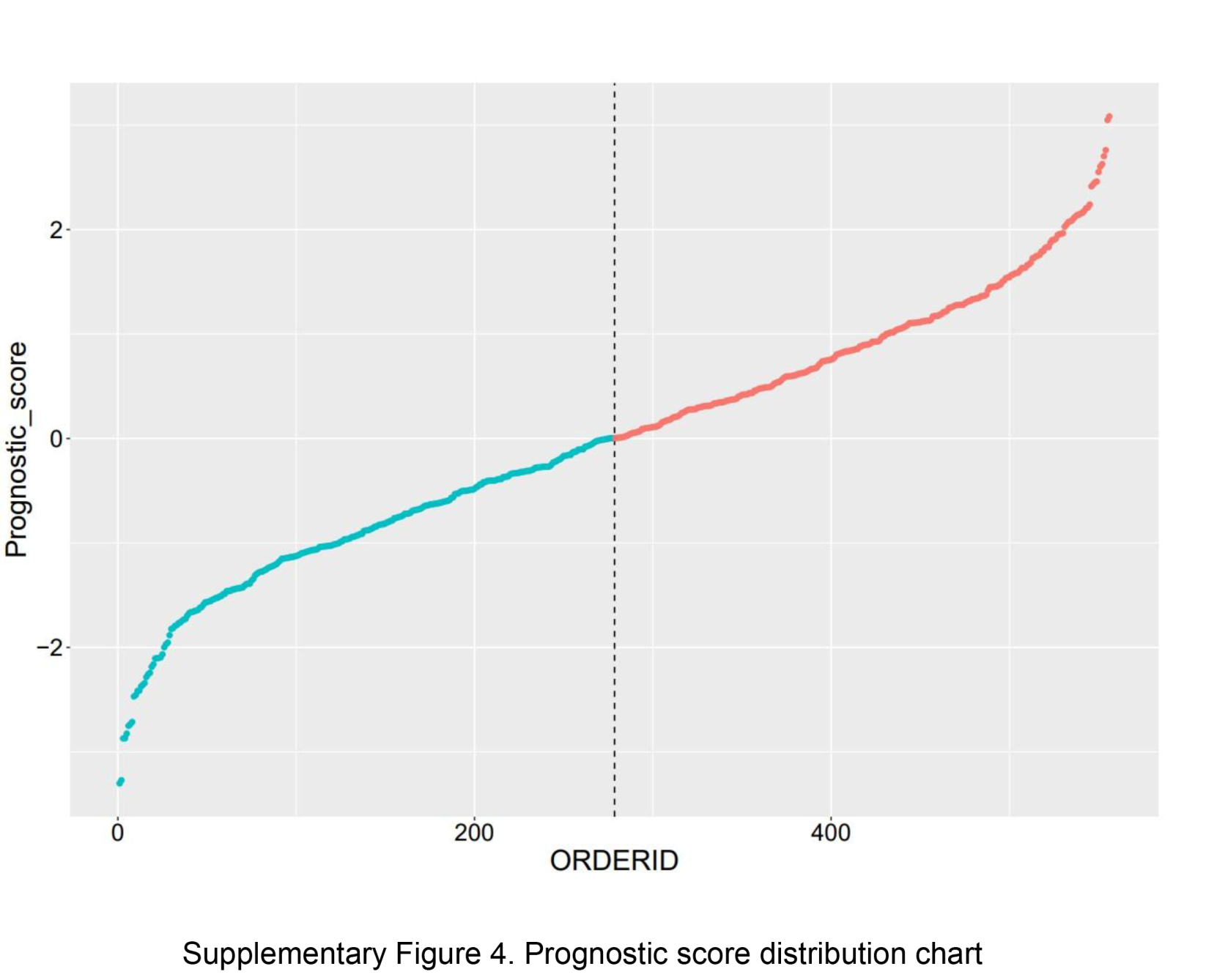


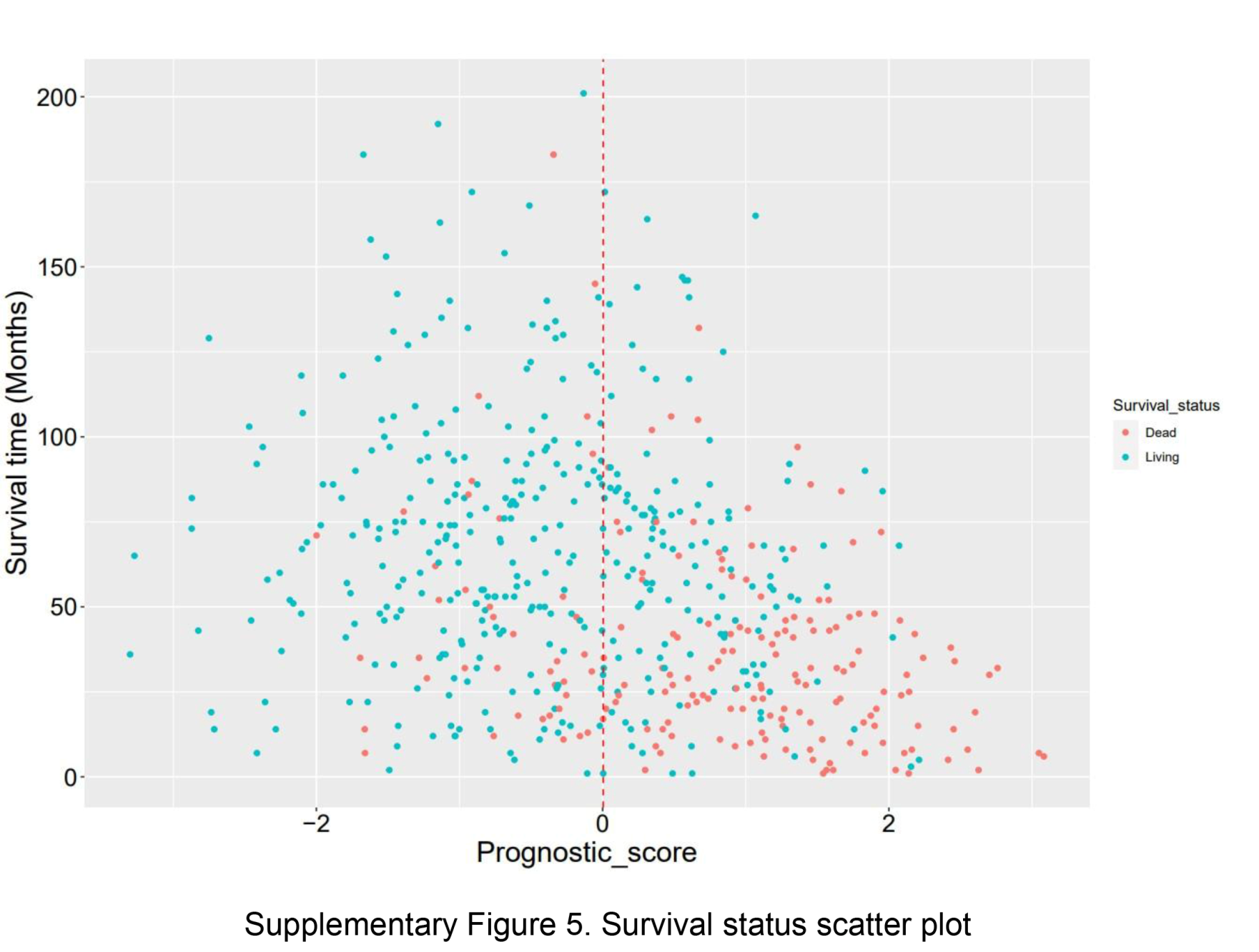


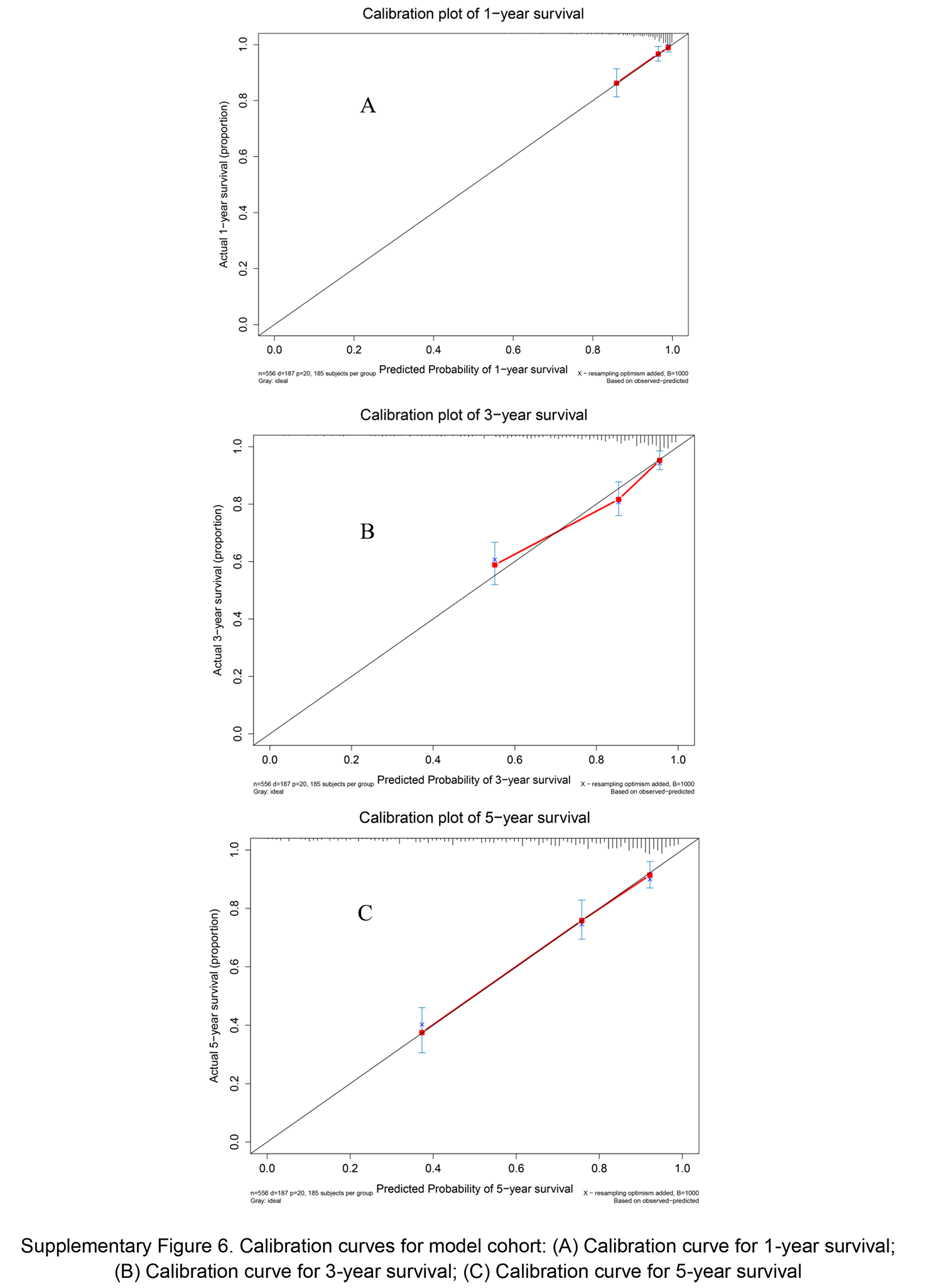


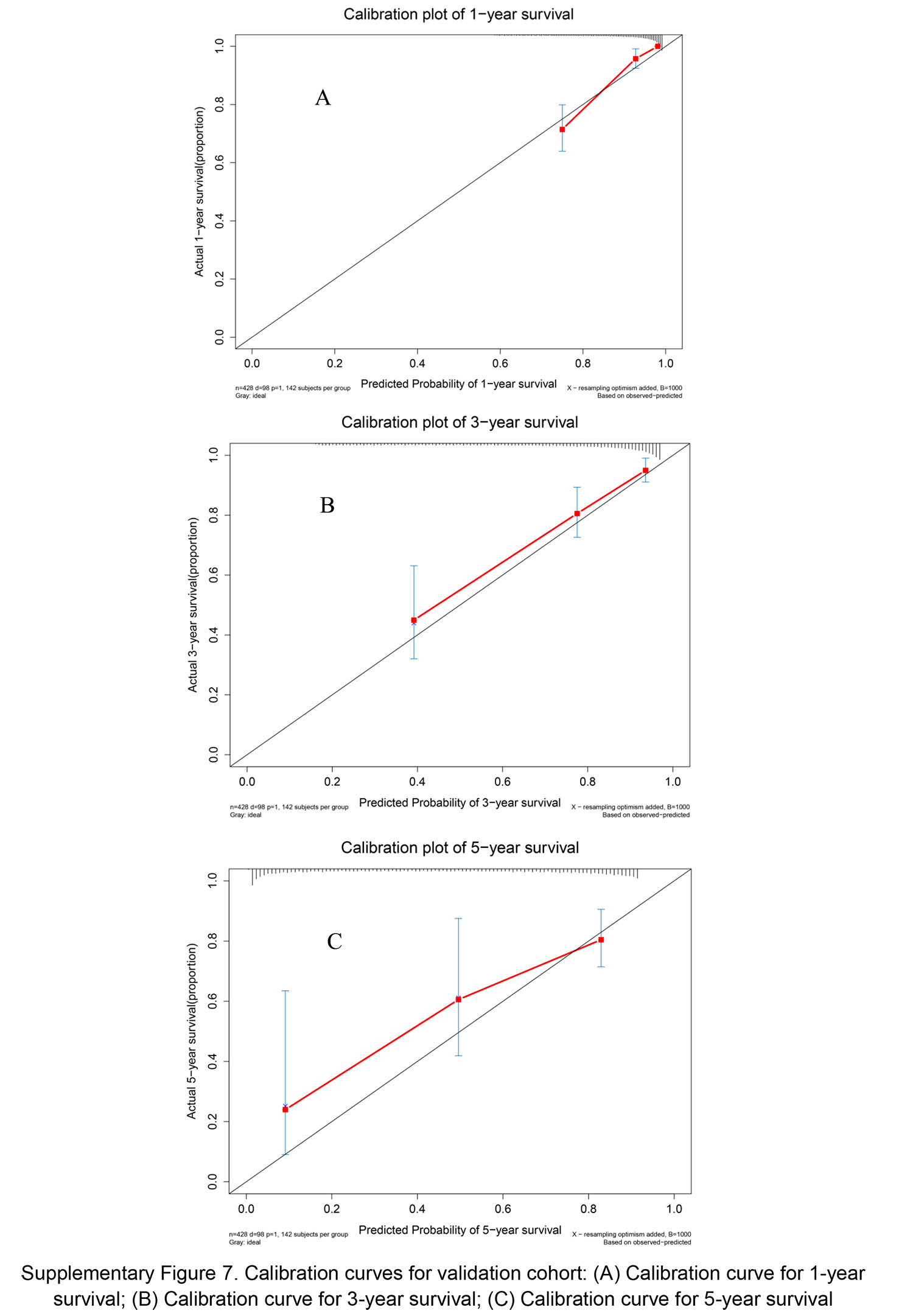


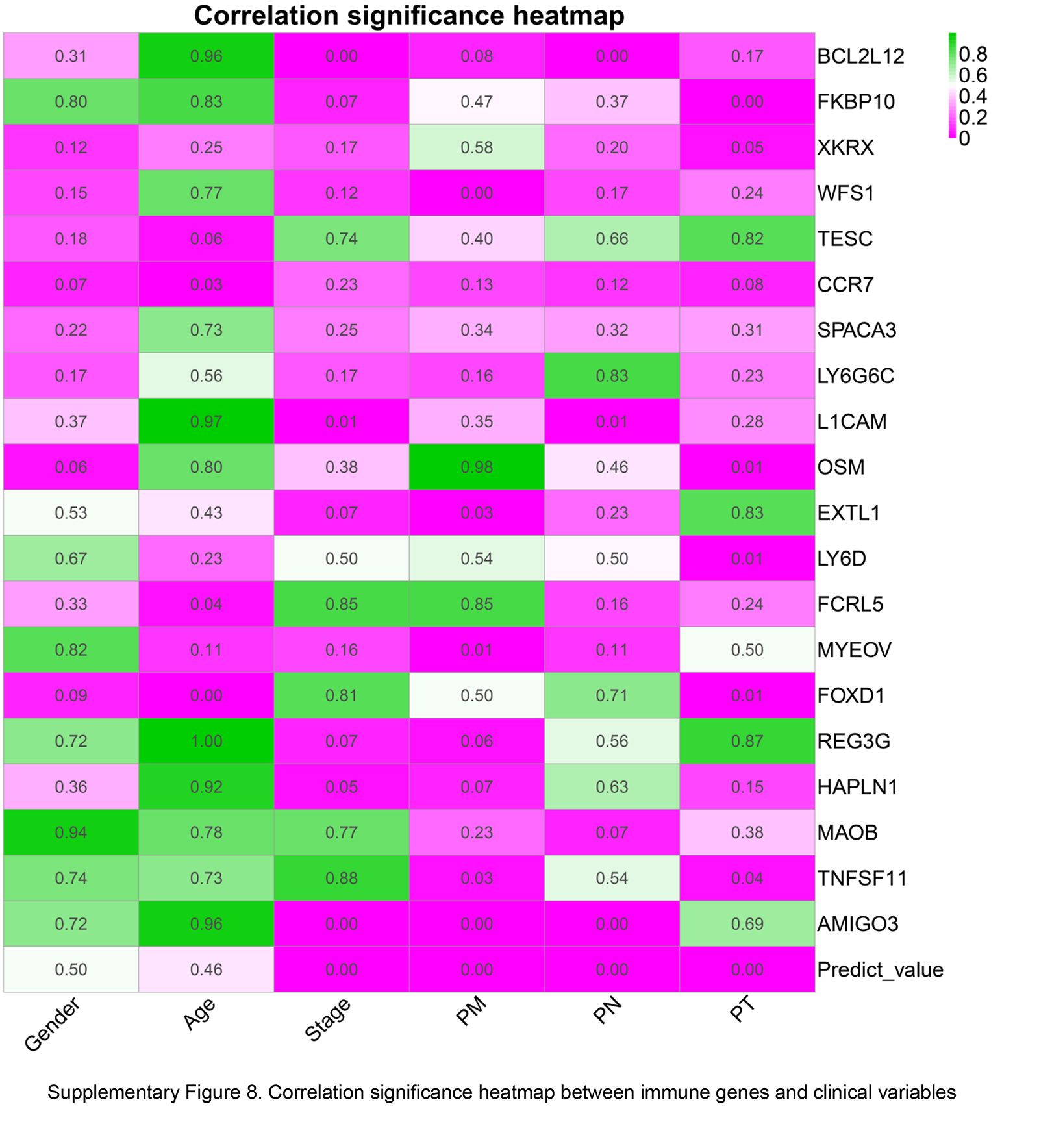


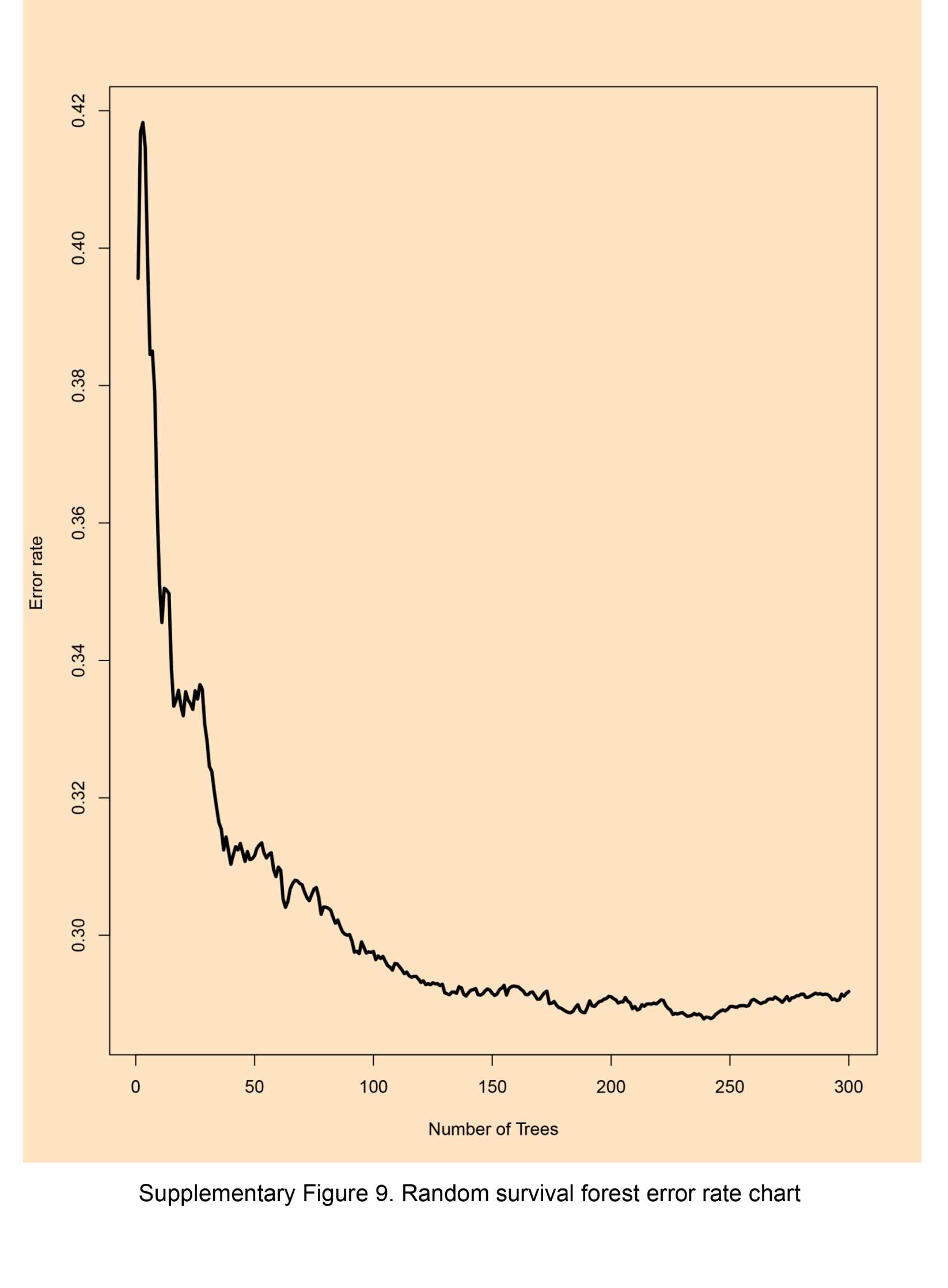


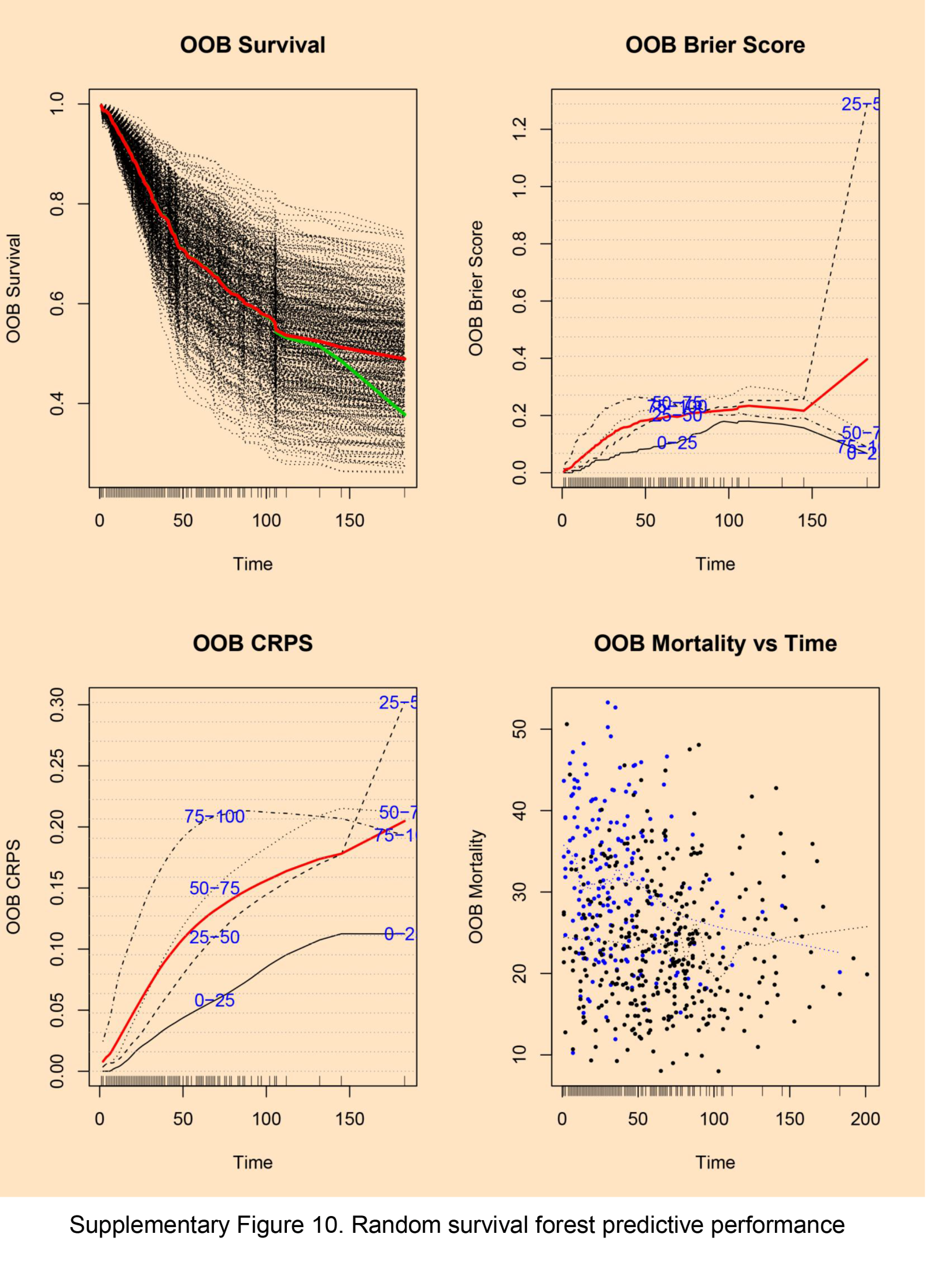


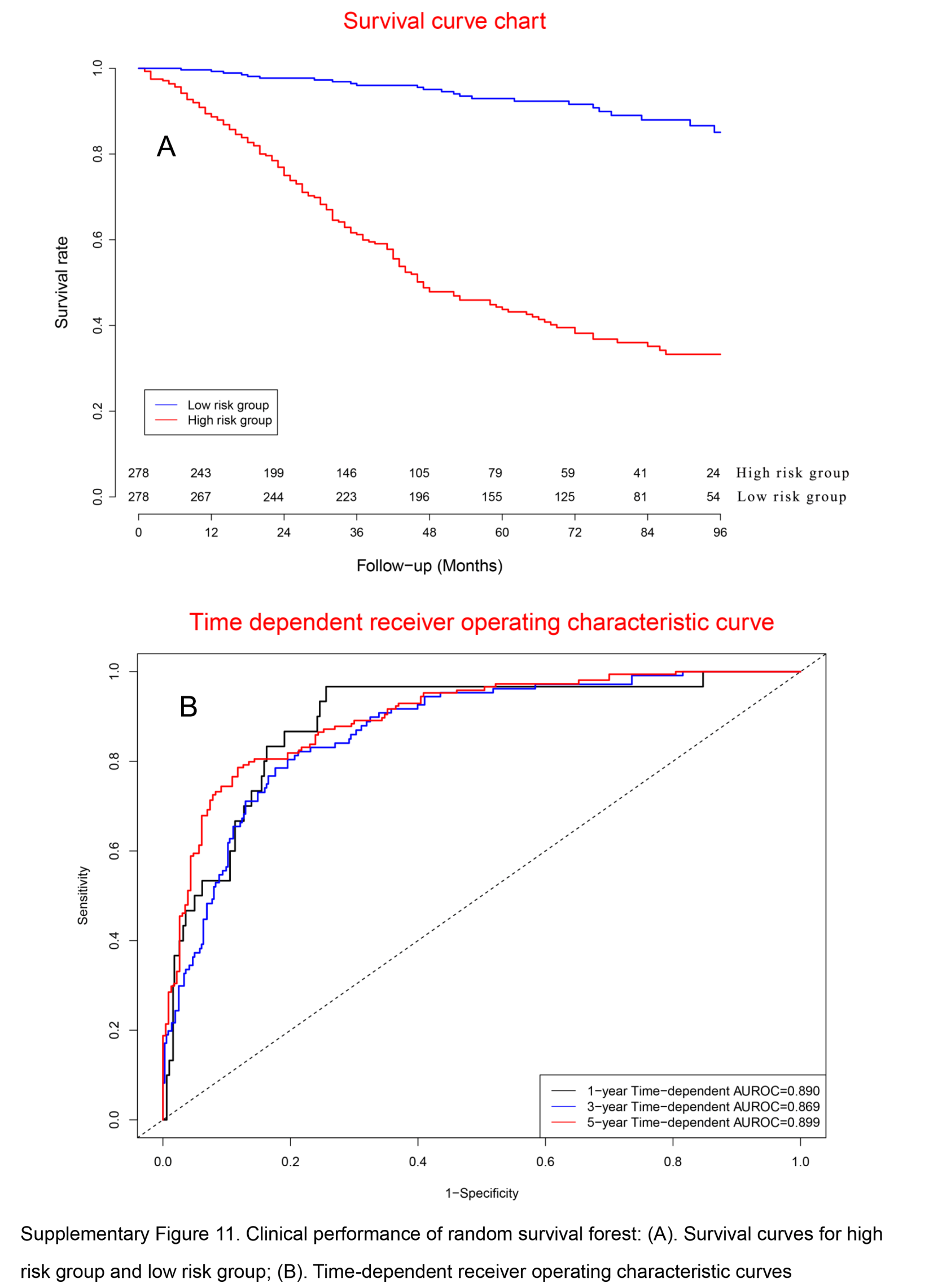


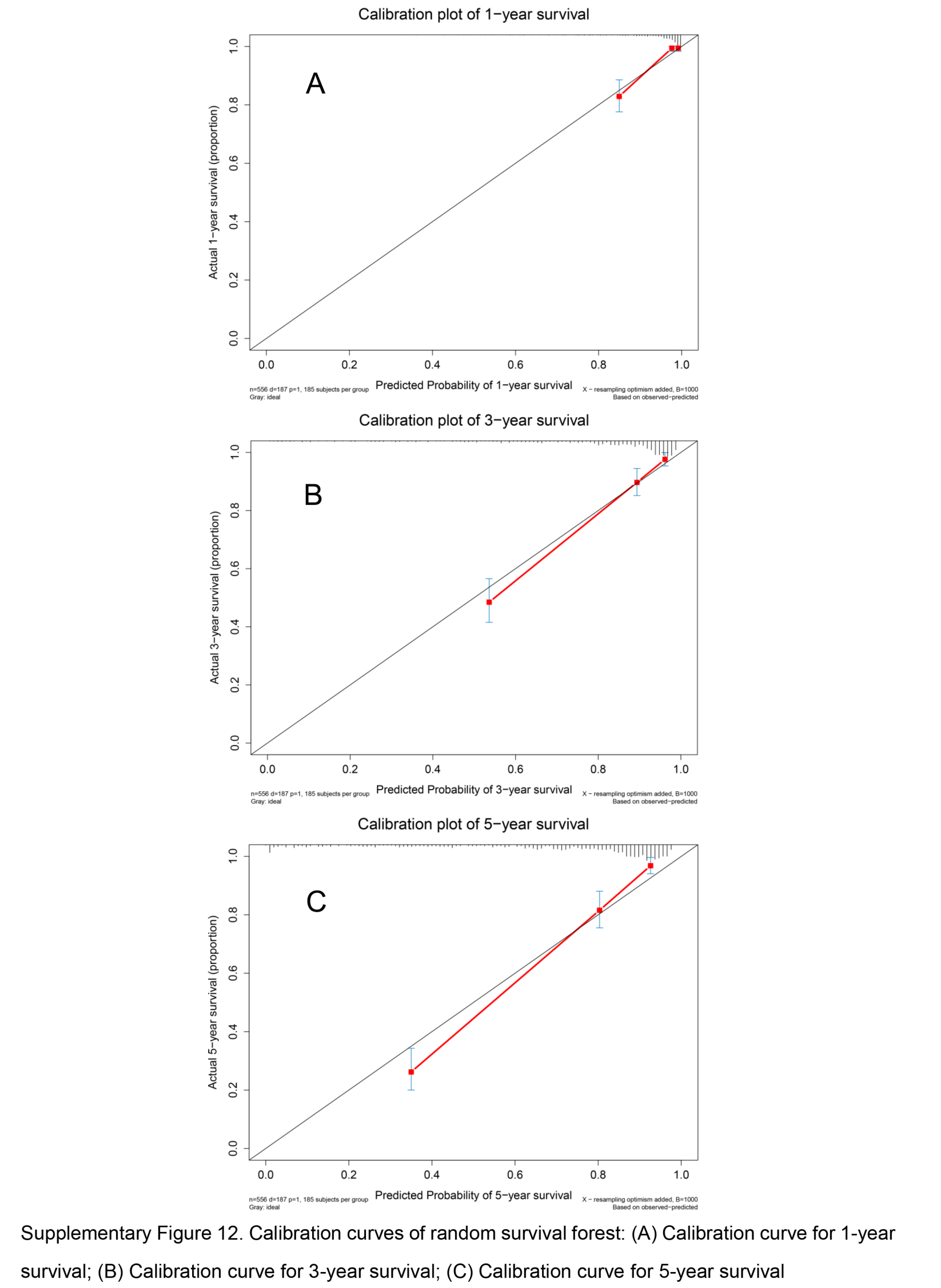


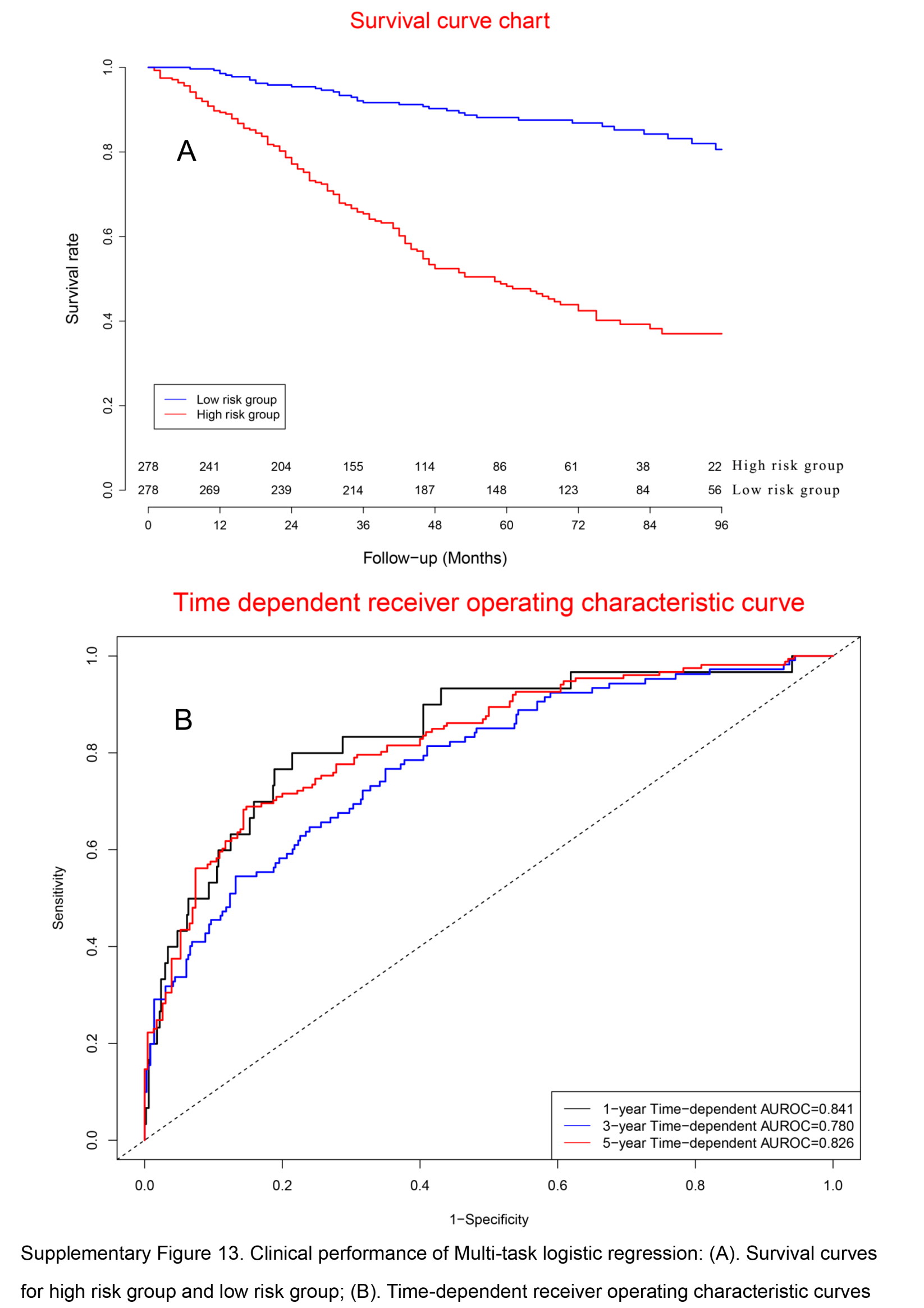


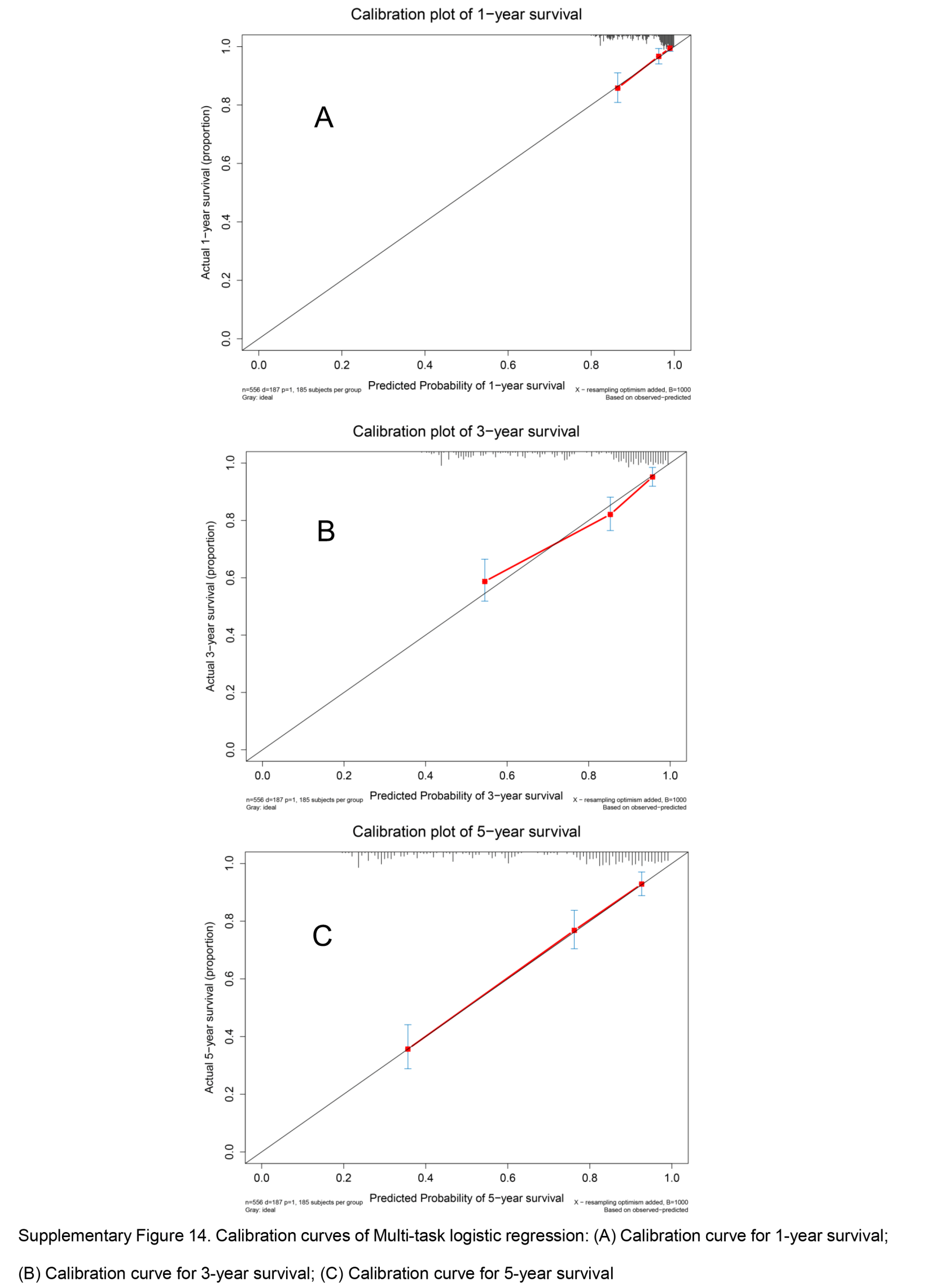


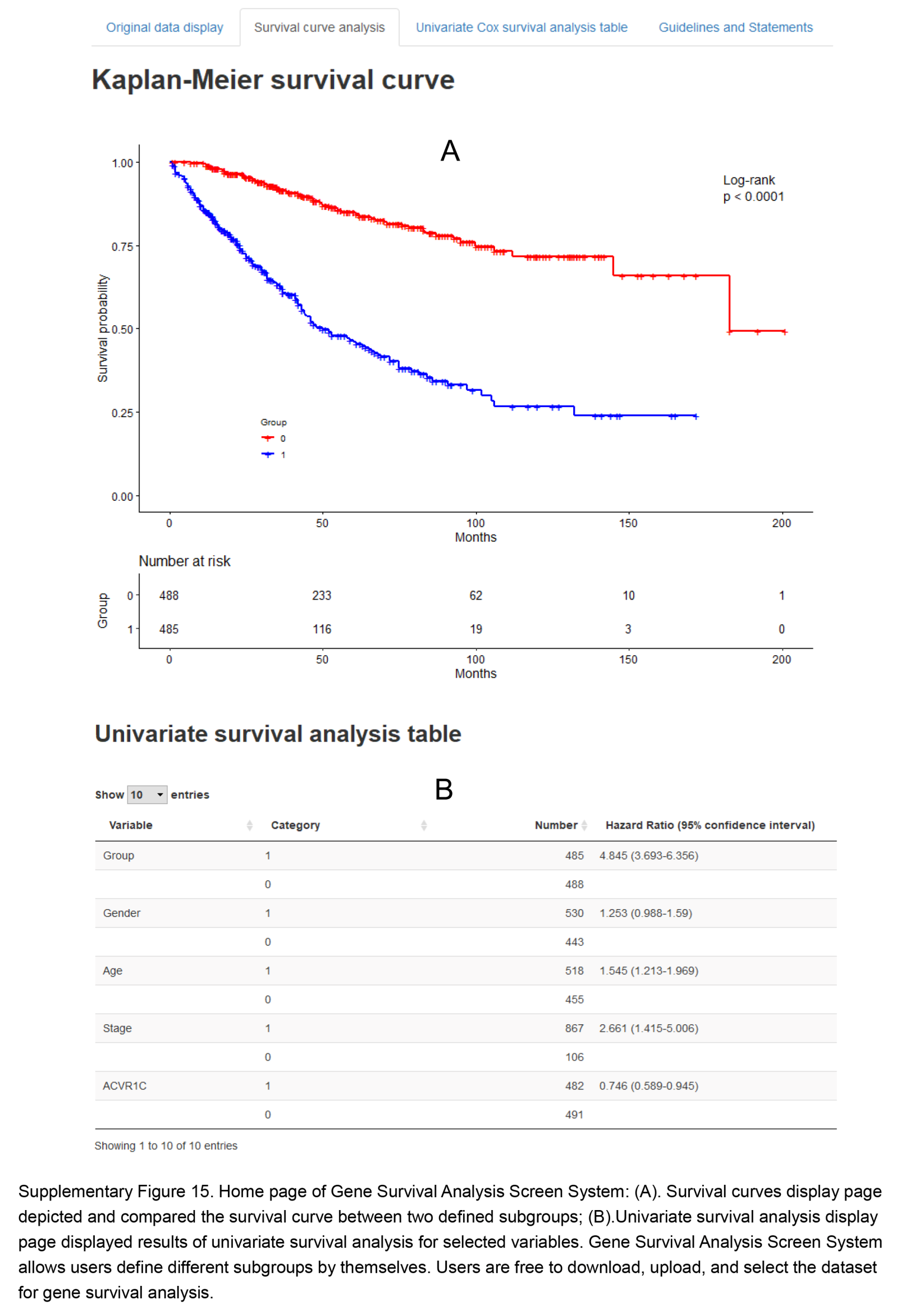

Supplement: Supplementary file 5 — Additional file 5. Supplementary Figure 1-15 (fifteen figures in total). [file 12859_2022_4657_MOESM5_ESM.docx]
